# Assessment of Undernutrition Among Under 5 Children in Developing Countries: A Systematic Review and Meta‐Analysis

**DOI:** 10.1002/hsr2.72087

**Published:** 2026-03-16

**Authors:** Mossamet Kamrun Nesa, Md. Rashed Babu, Sumaiya Tasnim, Md. Jamal Uddin

**Affiliations:** ^1^ Department of Statistics Shahjalal University of Science and Technology Sylhet Bangladesh; ^2^ Faculty of Graduate Studies Daffodil International University Dhaka Bangladesh

**Keywords:** childhood undernutrition, developing countries, stunting, underweight, wasting

## Abstract

**Background and Aim:**

Undernutrition is gradually declining worldwide except in sub‐Saharan Africa and parts of South Asia. Prevalence is almost stagnant in low‐and middle‐income countries (LIMCs), contributing to child mortality and disease burden. To systematically review existing literature and conduct a meta‐analysis to estimate the prevalence of childhood undernutrition—specifically stunting, wasting, and underweight—and identify associated factors among children under five in developing countries.

**Methods:**

Following PRISMA 2020 guidelines, this study systematically reviewed literature from 2010 to 2022 across PubMed, Scopus, Cochrane Library and Web of Science on developing countries. Study quality was assessed using JBI scores. Meta‐analyses estimated pooled prevalence and factors of undernutrition using the DerSimonian–Laird random‐effects model. Heterogeneity was assessed via *I*² and Cochran's Q. Subgroup analyses by year and continent were performed for undernutrition indicators. Sensitivity analyses showed minimal discrepancies, supporting the robustness of findings.

**Results:**

We included 157 studies in this research. Pooled prevalence of stunting, wasting, and underweight was 33% (95% CI: 31–35), 12% (95% CI: 11–13), and 23% (95% CI: 21–25), respectively. Subgroup analysis indicates that Asia had the highest wasting (13%, 95% CI: 11–16) and underweight (27%, 95% CI: 23–31), while Africa had the highest stunting (35%, 95% CI: 33–38. Significant heterogeneity was found across studies and continents (*p* < 0.05) for each indicator. Male children had 27% (POR:1.27; 95% CI: 1.22–1.32; *p *< 0.05), 31% (POR:1.31; 95% CI: 1.19–1.43; *p *= 0.08), and 44% (POR:1.44; 95% CI: 1.18–1.6; *p* < 0.05) higher odds of being stunted, wasted, and underweight. Rural residence, wealth status, parental education, child age group, mother BMI, breastfeeding, birth order, unprotected water source for drinking and food‐insecure household were key undernutrition factors.

**Conclusion:**

The findings underscore the need for targeted nutritional interventions addressing the identified socioeconomic and healthcare risk factors. Region‐specific strategies will be essential to sustainably reduce undernutrition.

## 1 Introduction

Childhood undernutrition has become an increasing public health concern worldwide, with a particular focus on developing nations. Undernutrition in under‐5 children is usually measured by three standard anthropometric indices: stunting, wasting, and underweight. According to the World Health Organization (WHO) report 2023, undernutrition is associated with about 2.7 million deaths every year among under‐5 children [1]. Roughly 45% of childhood fatalities worldwide were linked to undernutrition, with the majority occurring in low and middle‐income countries [2, 3]. Moreover, it is connected with an increased risk of all‐cause mortality as well as an increased risk of death from Diarrhea, Pneumonia, and Measles [4]. Globally, 155 million under‐5 children are stunted, 52 million are wasted, and approximately 101 million are underweight [1]. The international community has established a Sustainable Development Goal (SDG‐2.2) and committed to reducing all forms of childhood malnutrition by 2030 [5]. If the countries can achieve the SDG goal by 2030, then 11 million childhood deaths can be prevented globally [6].

According to the UNICEF 2021 report, children with malnutrition is more frequent in Africa and Asia [7]. In 2022, Asia had 52% and Africa had 43% of stunted children respectively, with more than three‐quarters (75%) and 22% of all children under five suffering from wasting [8]. In South and Southeast Asia, being underweight has been regarded as a major issue [9], and in Africa, the number of stunted children is remarkably high. Also, Western Africa, Southern Asia, and Southeast Asia were found to have estimated prevalence rates of undernutrition that were much higher than the estimates for the world as a whole [10, 11]. Therefore, to achieve the Sustainable Development Goal, it is also required to determine the contributing variables that lead to undernutrition.

A substantial literature has assessed that the three key indicators of undernutrition, that is, stunting (height‐for‐age) or wasting (weight‐for‐height) or underweight (weight‐for‐age) have a significant impact on child development [12, 13, 14, 15, 16]. Stunting and wasting are mostly responsible for child mortality in many developing countries [17], and malnutrition significantly affects childhood disability [18, 19]. Primary factors contributing to malnutrition in developing countries include Diarrhea, Acute Respiratory Infections (ARI), and Malaria [20].

Studies have identified various factors, such as household food insecurity, socioeconomic status, limited maternal education, early childhood development, and residence in rural areas, as primary correlates of child undernutrition [21, 22, 23, 24]. Educating mothers about starting and continuing breastfeeding is a key factor in improving an under‐five nutritional status. Additionally, promoting education and income‐generating activities within impoverished households is essential [25, 26]. Better access to essential maternal health services, delivery in a medical institution or with a qualified labor support professional, and improved sanitation infrastructure contributed to significantly improved child development [15]. There are large disparities in child undernutrition across genders, geographical regions, and economic groups [27]. The occurrence of malnutrition was significantly greater in children born with low birth weight compared to those with typical birth weights [28]. Furthermore, children's health and prospects are threatened in every country by climate change, ecological degradation, population displacement, violence, persistent inequality, and exploitative business practices [29].

A study for LMICs conducted a systematic review and showed an association between child undernutrition and responsive feeding [30]. In another study, O. R. Katoch prevailed in determinants of child malnutrition by using a systematic review [31]. Although notable number of studies have been done to assess childhood undernutrition and associated factors concentrating on specific regions, it is crucial to understand the generalized phenomenon related to factors regarding child undernutrition to consider the most effective solutions in developing countries to reduce the problem significantly. Some similar research with systematic review and meta‐analysis did not take into consideration all emerging nations. However, they only concentrate on specific regions. Also, some studies only address systematic reviews. Moreover, undernutrition remains a major public health concern in low‐ and middle‐income countries (LMICs), particularly among children under five. While numerous studies have addressed stunting, wasting, and underweight, evidence on the pooled odds ratios (PORs) for associated factors remains limited and fragmented. Despite existing interventions, gaps persist in understanding the true prevalence and key determinants of undernutrition. This necessitates robust and focused analyses to guide more effective and context‐specific strategies.

This study's strength lies in its comprehensive consideration of all possible emerging nations to identify associated factors, while simultaneously conducting meta‐analyses to illustrate the overall prevalence and PORs of these factors for three key nutritional indicators—stunting, wasting, and underweight—separately. Such an approach is intended to draw the attention of policymakers worldwide. The integration of both systematic review and meta‐analysis adds credibility to the findings. The outcomes of this study aim to strengthen current child nutrition strategies and interventions, enabling policymakers to better assess progress toward achieving SDGs 1, 2, and 3. Therefore, the overall objective of this study was to systematically review existing literature and conduct a meta‐analysis to estimate the prevalence of childhood undernutrition—specifically stunting, wasting, and underweight—and identify associated factors among children under five in developing countries.

## 2 Methods

The protocol for this systematic review and meta‐analysis was registered with the International Prospective Register of Systematic Reviews (PROSPERO) under registration number CRD42024538797. This systematic review adhered to the 2020 Reporting Items for Systematic Reviews and Meta‐Analysis (PRISMA) [32] guidelines during its execution. The PRISMA checklist for abstract (Appendix Table S1) and manuscript (Appendix Table S2) are attached in the Supplementary files.

### 2.1 Search Strategy

**FIGURE 1:**
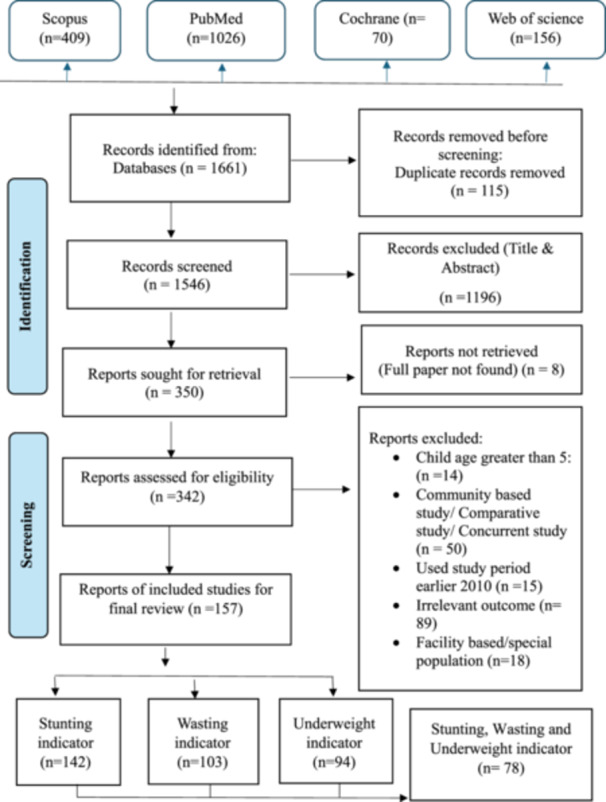
Flow chart of systematic review and data screening.

A compilation of relevant MeSH terms and subheadings for keywords was created and utilized to thoroughly search peer‐reviewed articles across four computerized bibliographic databases, which include: Scopus, PubMed, Cochrane Database, and Web of Science (Figure 1). The search keywords were (“prevalence” or “percentage” or “incidence”) and (“stunting” or “wasting” or “underweight” or “malnutrition” or “undernutrition” or “nutritional status” or “protein‐energy malnutrition” or “child nutrition disorder” or “infant nutrition disorder”) AND (“child” or “under‐five” or “under‐five‐children” or “preschool” or “pediatric” or “infant”).

### 2.2 Inclusion and Exclusion Criteria

The chosen research focused on children under 5 years old, investigating factors linked to child undernutrition, which includes stunting, wasting, underweight, and overall nutritional status. The studies were conducted between 2010 and 2022, following observational methodologies, and were published in English‐language peer‐reviewed journals. Excluded from consideration were conference abstracts, unpublished studies, and individuals older than 5 years, including adolescents and adults. Appendix Table S3 presents the inclusion and exclusion criteria for this study. To address risk factors, we included only studies with statistically significant adjusted odds ratios in the final analysis.

### 2.3 Data Extraction

Data from each study were systematically categorized and stored, covering diverse aspects such as authors' names, publication year, continent, specific location (country), research design, sample size, data source, and prevalence of indicators listed in Appendix Table S4. Furthermore, demographic characteristics, socioeconomic factors, information concerning both the father and mother, as well as health and nutritional data for both the child and mother, were extracted mainly through studies that examined the prevalence of these indicators. We included only studies that reported adjusted odds ratios. Among these, only statistically significant adjusted estimates were considered in the final synthesis. This comprehensive storage system included nutritional indicator measures like prevalence with standard deviation, odds ratios (OR), hazard ratios (HR), and relative risks (RR), each accompanied by a 95% confidence interval. Notably, these measures were analyzed separately for stunting, wasting, and underweight.

### 2.4 Quality Assessment

The reviewers evaluated the quality of the pertinent studies included in Appendix Table S5 using the Joanna Briggs Institute (JBI) [33] critical appraisal score specifically designed for analytical cross‐sectional studies. The assessment was recorded using Microsoft Excel. The score depends on the appraisal category (yes, no, unclear, and not applicable). Studies score 8 if all the criteria are met properly and zero if none of the requirements are met [34]. The scale has eight checklists to evaluate the relevancy and determine the final quality score, which is as follows: High‐quality papers score 7 to 8 on the checklist, medium‐quality papers score between 6 and 7, and low‐quality papers score less than 6.

Two reviewers, MR Babu and S Tasnim reviewed the retrieved publication and screened all studies separately to maintain the relevancy of the articles. After removing duplicates, the first phase of screening includes a title review, and the second phase is conducted through an abstract review. The eligible studies were included in the final screening after full‐text reading.

### 2.5 Reliability Assessment

Throughout the process, the suitability of the articles was assessed at each stage, and any inconsistencies and disagreements were addressed. MK Nesa and MJ Uddin were available to provide resolution concerning the final selection of publications for inclusion.

### 2.6 Outcome Measures

The primary outcomes of the study are the prevalence of child nutritional indicators, namely stunting, wasting, and underweight, as well as identifying changes in nutritional status from 2010 to 2022 and determining the associated risk factors that significantly affect the child's nutritional status. Children with height‐for‐age, weight‐for‐age, and weight‐for‐height scores below −2 standard deviations (SD) were categorized as stunted, underweight, and wasted, respectively, based on WHO 2006 standards [35]. If a characteristic was identified as a contributing factor for childhood undernutrition in four or more studies was considered for inclusion in the meta‐analysis; otherwise, it was excluded. The primary emphasis was on prevalence and key risk indicators such as OR, HR, and RR, each paired with a 95% confidence interval.

### 2.7 Sensitivity Analysis

To assess the robustness of the outcomes of this study, we examine the presence of outliers. Upon their removal, we reassess the results in their entirety. Consequently, minimal disparities among the outcomes indicate the robustness of the study.

### 2.8 Statistical Analysis

The study retrieved relevant articles from a systematic review considering the measurements of three indicators. The metrics are displayed utilizing mean or prevalence in these articles. Therefore, meta‐analysis integrates studies with prevalence rates and excludes studies that consider mean *Z*‐score of indicators, in order to maintain consistent and reliable evaluation. Consequently, the final analysis includes the prevalence rates of stunting, wasting, and underweight.

The main emphasis of this meta‐analysis was to ascertain the effect size, such as the combined prevalence, indicated by the proportion of children under the age of five affected by stunting, wasting, and underweight. We conducted subgroup analyses by publication year and continent to evaluate the prevalence of stunting, wasting, and underweight separately. The POR was determined using the effect size, which combines the odds of multiple studies and finds the overall effect. We assessed variability among studies using both the *I*‐squared statistic and Cochran's *Q* statistic, along with the corresponding *p*‐value indicating heterogeneity (right‐tailed). Aggregate estimates were calculated using the DerSimonian–Laird random‐effects model and presented as proportions and odds ratios with their respective 95% confidence intervals. We followed the SAMPLE guidelines for reporting risk, rates, and ratios.

To identify potential publication bias, we employed both visual inspection of funnel plots and Egger's tests, with a particular focus on detecting funnel plot asymmetry [36]. We considered statistical significance at a threshold of *p* < 0.05 (two‐tailed). Additionally, we utilized the online literature management tool Mendeley to organize the articles. Meta‐analyses were conducted using STATA 16.0 software (STATA Corp.).

## 3 Results

### 3.1 Systematic Review

This study identified a total of 1661 articles through the database search after removing 115 duplicates. 1546 articles were included in the title and abstract screening, of which 342 met the eligibility criteria for assessment. A further 185 studies were excluded based on criteria such as child age, study design, period of study, facility‐based or special population, and outcome measures. Following completion of the review and screening process, 157 studies remained for the final review, with 78 studies covering all three undernutrition indicators (stunting, wasting, and underweight). Specifically, we found that 142 studies addressed stunting, 103 studies addressed wasting, and 94 studies addressed underweight. Figure 1 illustrates the study selection and screening process. Tables 2, 3, and 4 present the number of studies corresponding to each risk factor.

### 3.2 Quality Assessment

The study found 55 papers scored above 7, 99 papers scored between 5 and 6, and 3 papers scored below 6 according to the JBI quality review. Therefore, the overall appraisal was 35.0% of the papers classified as high quality, and 63.1% are in medium quality. Only 1.9% of the paper did not meet the desirable criteria (Appendix Table S5).

### 3.3 Meta‐Analysis

Since the meta‐analysis solely considers studies with prevalence rate, subsequently the final analysis comprises 142 articles excluding 15 articles out of 157 papers, all of which provide prevalence of stunting; out of 105 articles, 102 articles examine prevalence of wasting, and 93 articles address prevalence of underweight.

Based on 142 selected articles, we found the pooled prevalence of stunting in developing countries was 32.6% (95% CI: 30.7–34.5, *I* ^2^ = 99.8, *p *< 0.05, *n* = 141) (see Table 1). According to subgroup analysis by continent, the maximum pooled prevalence of stunting was found in the Africa continent at 34.9% (95% CI: 32.6–37.2, *p *< 0.05, *n* = 80), the second leading pooled prevalence was found in the Asia continent at 30.5% (95% CI: 27.4–33.7, *n* = 56) followed by South America had 14.8% (95% CI: 13.1–16.5, *n* = 3) of stunted children, respectively (Appendix Figure S1). Tests of heterogeneity indicate (*Q* = 427.83, *p* < 0.5) significant heterogeneity presents between studies. Subgroup analysis by year revealed that the prevalence of stunting fluctuated between 22.1% [95% CI: 11.4–32.8, *p *< 0.05, *n* = 5] and 39.6% [95% CI: 34.4–44.8, *p *< 0.05, *n* = 1] from 2010 to 2022 (*Q* = 15.82, *p *= 0.11) (see Table 1). In this study, 102 of the studies fulfilled all the inclusion criteria for wasting. The pooled prevalence of wasting in developing countries was found 11.5% (95% CI: 10.2–12.9, *I* ^2^ = 99.5, *p *< 0.05, *n* = 102). Subgroup analysis by continent illustrates the maximum prevalence of wasting in the Asia continent at 13.2% (95% CI: 10.7–15.6, *p* <0.05, *n* = 40), and in the Africa continent, it was found 10.6% (95% CI: 9.1–12.2, *p *< 0.05, *n* = 60) (Appendix Figure S2) respectively. The Cochrane *Q* test found significant (*p* < 0.5) and indicates that heterogeneity presents between studies. Subgroup analysis by year found the prevalence of wasting fluctuated from 7.4% [2014: 95% CI: 1.2–13.6, *p *< 0.05, *n* = 4] to 16.8% [2013: 95% CI: 5.3–28.3, *p *< 0.05, *n* = 3] (*Q* = 6.58, *p* = 0.77) from 2012 to 2022 (see Table 1). For underweight, 93 studies covered all the inclusion criteria. From reviewed studies, the pooled prevalence of underweight in developing countries was found 22.6% (95% CI: 20.4–24.9, *Q* = 60388.6, *I* ^2^ = 99.78, *p *< 0.05, *n* = 93). Subgroup analysis by continent in Asia, the prevalence of being underweight was found 26.1% (95% CI: 22.1–30.0, *p* < 0.05, *n* = 40), in Africa, it was found 20.1% (95% CI: 17.7–22.5, *p* < 0.05, *n *= 52), and for South America found 17.3% (95% CI: 16.2–18.4, *p* < 0.05, *n* = 1) (Appendix Figure S3). Test of *Q* value (*Q* = 20.36, *p* < 0.5) indicates there presents significant heterogeneity between studies. Subgroup analysis by year found underweight fluctuation from 7% (95% CI: 2.9–11.0, *p *< 0.05, *n* = 3) to 32.1% (95% CI: 27.1–37.1, *p* < 0.05, *n* = 1) [*Q* = 75.88, *p* < 0.05] from 2011 to 2022. We visually inspected the funnel plot for asymmetry of stunting, wasting, and underweight. The funnel plots for stunting (Appendix Figure S4), wasting (Appendix Figure S5), and underweight (Appendix Figure S6) displayed notable irregularities, indicating potential publication bias. Likewise, the Egger test uncovered a significant intercept (*p *< 0.05), reinforcing the indication of publication bias.

**TABLE 1:** Pooled prevalence of childhood undernutrition in developing countries (2010–2022), with subgroup analysis by continent and year.

Indicators	No. of studies	Prevalence (95% CI)	*p* value	Heterogeneity			Egger's test (*p*‐value)
				*Q*‐value	*p* value	*I* ^2^	
Stunting	141	32.6 (30.7–34.5)	< 0.05	75,504.9	< 0.05	99.8	< 0.05
**Subgroup analysis of Stunting for Continent**							
Africa	80	34.9 (32.6–37.2)	< 0.05	13,502.2	< 0.05	99.3	
Asia	56	30.5 (27.4–33.7)	< 0.05	55,361.1	< 0.05	99.9	
North America	1	11.9 (10.2–13.6)	< 0.05	0.0	—	—	
Oceania	1	37.1 (33.9–40.3)	< 0.05	0.0	—	—	
South America	3	14.8 (13.1–16.5)	< 0.05	9.62	< 0.05	75.7	
**Subgroup analysis of Stunting for Year**							
2012	1	39.6 (34.4–44.8)	< 0.05	—	—	—	
2013	3	29.6 (14.3–45.0)	< 0.05	51.8	< 0.05	98.2	
2014	5	22.1 (11.4–32.8)	< 0.05	239.9	< 0.05	98.7	
2015	10	38.0 (30.5–45.6)	< 0.05	611.5	< 0.05	98.9	
2016	5	36.3 (30.6–42.0)	< 0.05	236.9	< 0.05	98.1	
2017	15	35.5 (31.3–39.7)	< 0.05	867.5	< 0.05	98.7	
2018	13	32.1 (26.7–37.5)	< 0.05	948.2	< 0.05	98.1	
2019	25	31.3 (26.8–37.1)	< 0.05	7619.6	< 0.05	99.7	
2020	25	31.9 (26.8–37.1)	< 0.05	24,506.9	< 0.05	99.9	
2021	25	30.8 (25.7–35.9)	< 0.05	12,591.3	< 0.05	99.8	
2022	14	35.3 (29.8–40.8)	< 0.05	2429.9	< 0.05	99.8	
Wasting	102	11.5 (10.2–12.9)	< 0.05	16,180.9	< 0.05	99.5	< 0.05
**Subgroup analysis of Wasting for Continent**							
Africa	60	10.6 (9.1–12.2)	< 0.05	5303.0	< 0.05	99.3	
Asia	40	13.2 (10.7–15.6)	< 0.05	10,525.5	< 0.05	99.7	
Oceania	1	4.0 (2.7–5.3)	< 0.05	—	—	—	
South America	1	7.0 (4.4–9.6)	< 0.05	—	—	—	
**Subgroup analysis of Stunting for Year**							
2012	1	11.3 (7.9–14.7)	< 0.05	0.0	< 0.05	—	
2013	3	16.8 (5.3–28.3)	< 0.05	42.2	< 0.05	97.9	
2014	4	7.4 (1.2–13.6)	< 0.05	68.2	< 0.05	98.3	
2015	7	12.6 (8.1–17.2)	< 0.05	185.1	< 0.05	96.5	
2016	4	8.8 (3.4–14.1)	< 0.05	215.2	< 0.05	98.9	
2017	10	14.3 (9.3–19.3)	< 0.05	1067.6	< 0.05	99.4	
2018	7	10.8 (1.0–20.4)	< 0.05	761.8	< 0.05	99.7	
2019	20	10.1 (7.7–12.4)	< 0.05	2567.4	< 0.05	99.4	
2020	17	11.0 (8.4–13.7)	< 0.05	2341.1	< 0.05	99.3	
2021	20	12.6 (9.2–15.9)	< 0.05	2767.9	< 0.05	99.7	
2022	9	11.5 (7.8–15.2)	< 0.05	1544.4	< 0.05	98.7	
Underweight	93	22.6 (20.4–24.9)	< 0.05	60,388.6	< 0.05	99.8	0.121
**Subgroup analysis of Underweight for Continent**							
Africa	52	20.1 (17.7–22.5)	< 0.05	8848.0	< 0.05	99.4	
Asia	40	26.1 (22.1–30.0)	< 0.05	49,641.2	< 0.05	99.9	
South America	1	17.3 (16.2–18.4)	< 0.05	—	—		
**Subgroup analysis of Underweight for Year**							
2012	1	32.1 (27.1–37.1)	< 0.05	—	—	—	
2013	3	7.0 (2.9–11.0)	< 0.05	18.2	< 0.05	91.6	
2014	3	18.0 (7.6–43.5)	0.168	136.9	< 0.05	99.8	
2015	6	21.7 (14.5–28.9)	< 0.05	225.2	< 0.05	97.1	
2016	4	26.1 (17.2–35.1)	< 0.05	299.4	< 0.05	99.2	
2017	9	24.4 (17.8–30.9)	< 0.05	942.6	< 0.05	99.3	
2018	10	25.0 (16.0–34.0)	< 0.05	2511.1	< 0.05	99.8	
2019	17	18.0 (13.8–22.3)	< 0.05	7573.6	< 0.05	99.7	
2020	15	23.5 (19.3–27.7)	< 0.05	7546.3	< 0.05	99.7	
2021	19	24.3 (19.7–29.0)	< 0.05	12,892.9	< 0.05	99.7	
2022	6	29.6 (19.4–39.8)	< 0.05	734.2	< 0.05	99.6	

The pooled risk factors (POR) related to stunting in developing countries are presented in Table 2. Male children found higher stunted; POR: 1.26 (95% CI: 1.21–1.31; *I* ^2^ = 0.0, *p* < 0.05, *n* = 13), whereas female child were found 18% less likely stunted (OR: 0.82, 95% CI: 0.79–0.88; *I* ^2^ = 93.6, *p* = 0.97, *n* = 22), and those from rural residences were more stunted (POR: 1.52; 95% CI: 1.21–1.83; *I* ^2^ = 99.14, *p* < 0.05, *n* = 18) respectively. Children from the poorest families had the highest odds of being stunted (POR: 2.84, 95% CI: 2.16–3.52; *I* ^2^ = 92.85, *p* <0.05, *n* = 16). Additionally, children from poor (POR: 1.77, 95% CI: 1.25–2.29; *I* ^2^ = 99.1, *p* < 0.05, *n* = 19) and middle‐wealth index families (POR: 1.16, 95% CI: 0.94–1.39; *I* ^2^ = 97.79, *p* <0.05, *n* = 26) were more likely to be stunted (POR > 1) compared to children from rich and richest families. A higher chance of stunting was found (POR > 1) if mothers had no education (illiterate) (POR: 2.45, 95% CI: 1.77–3.13; *I* ^2^ = 94.1, *p *< 0.05, *n* = 17), and less education. Similarly, a higher chance of stunting was found if the father was illiterate, POR: 2.17 (95% CI: 1.77–2.57; *I* ^2^ = 0.0, *p *= 0.276, *n* = 5).

**TABLE 2:** Summary of meta‐analysis for associated factors of childhood stunting in developing countries (2010–22).

Factors	No. of studies	POR (95% CI)	*p* value	Heterogeneity			Egger's test (*p*‐value)
				*Q*‐value	*p* value	*I* ^2^	
**Child Sex**							
Male	13	1.26 (1.21–1.31)	< 0.05	10.41	0.580	0.00	< 0.05
Female	22	0.82 (0.76–0.88)	< 0.05	199.3	< 0.05	93.58	0.970
Residence: Rural	18	1.52 (1.21–1.83)	< 0.05	309.11	< 0.05	99.14	< 0.05
**Wealth index**							
Poorest	16	2.84 (2.16–3.52)	< 0.05	161.4	< 0.05	92.85	< 0.05
Poor	19	1.77 (1.25–2.29)	< 0.05	268.04	< 0.05	99.11	< 0.05
Middle	26	1.16 (0.94–1.39)	< 0.05	226.0	< 0.05	97.79	< 0.05
Rich	15	0.97 (0.69–1.24)	< 0.05	118.1	< 0.05	97.43	< 0.05
Richest	19	0.76 (0.55–0.97)	< 0.05	229.2	< 0.05	97.77	< 0.05
**Mother education**							
Illiterate	17	2.45 (1.77–3.13)	< 0.05	163.9	< 0.05	94.11	< 0.05
Primary	29	1.15 (0.94–1.36)	< 0.05	146.78	< 0.05	98.24	< 0.05
Less than SSC	6	1.12 (0.76–1.49)	< 0.05	44.06	< 0.05	92.64	0.09
SSC	21	0.86 (0.68–1.04)	< 0.05	352.81	< 0.05	99.13	< 0.05
SSC and above	5	0.69 (0.61–0.78)	< 0.05	5.32	0.256	32.33	0.618
HSC and above	9	0.39 (0.29–0.49)	< 0.05	213.5	< 0.05	97.3	0.52
**Father education**							
Illiterate	5	2.17 (1.77–2.57)	< 0.05	2.29	0.683	0.0	0.276
Primary	7	1.51 (1.02–2.01)	< 0.05	84.26	< 0.001	93.65	0.097
SSC	7	1.03 (0.61–1.46)	< 0.05	39.81	< 0.05	96.51	< 0.05
**Child age group**							
6 to 11	13	1.57 (1.06–2.08)	< 0.05	184.73	< 0.05	95.41	< 0.05
12 to 23	31	2.67 (2.16–3.17)	< 0.05	1514.4	< 0.05	98.53	< 0.05
24 to 35	22	3.31 (2.56–4.06)	< 0.05	282.96	< 0.05	98.82	< 0.05
36 to 47	17	3.16 (2.36–3.96)	< 0.05	225.65	< 0.05	98.71	< 0.05
48 to 59	18	2.42 (1.72–3.13)	< 0.05	195.5	< 0.05	98.92	< 0.05
**Child size at birth**							
Small	8	1.97 (1.54–2.39)	< 0.05	27.48	< 0.05	84.99	< 0.05
Average	9	1.07 (0.84–1.29)	< 0.05	88.83	< 0.05	95.30	0.055
Large	6	0.73 (0.54–0.91)	< 0.05	113.2	< 0.05	92.33	0.55
Low birth weight	3	1.95 (0.67–3.24)	< 0.05	8.10	< 0.05	78.02	0.22
**Mother's BMI status**							
Underweight	12	1.59 (1.29–1.89)	< 0.05	80.22	< 0.05	98.65	< 0.05
Normal	7	1.51 (1.15–1.87)	< 0.05	43.43	< 0.05	80.66	0.05
Overweight	7	0.86 (0.68–1.03)	< 0.05	102.45	< 0.05	96.02	0.15
**Mother's profession**							
Service holder	5	1.33 (1.19–1.47)	< 0.05	3.85	0.43	0.0	0.093
ANC Status: 1–3	6	0.83 (0.73–0.93)	< 0.05	26.44	< 0.05	88.48	0.22
Exclusive breastfeeding: Yes	4	0.66 (0.37–0.95)	< 0.05	41.47	< 0.05	99.17	0.25
**Family member**							
Greater than 5	5	1.24 (0.93–1.55)	< 0.05	15.19	< 0.05	97.61	< 0.05
**Water status**							
Unimproved	6	1.19 (1.08–1.32)	< 0.05	9.39	0.094	30.77	< 0.05
**Sanitation status**							
Improved	6	0.82 (0.74–0.89)	< 0.05	10.52	0.062	7.57	0.06
Unimproved	4	1.44 (1.07–1.81)	< 0.05	37.78	< 0.05	91.59	0.23
No. of children: 4	4	1.15 (0.42–1.87)	< 0.05	21.18	< 0.05	83.67	0.45
**Birth order**							
2–4	7	1.49 (1.04–1.94)	< 0.05	28.52	< 0.05	99.32	< 0.05
5 & above	5	1.33 (1.27–1.39)	< 0.05	2.88	0.577	0.0	0.12
Diarrhea	10	1.36 (1.19–1.52)	< 0.05	22.20	< 0.05	54.89	< 0.05
**Food security status**							
Severe	5	1.37 (1.24–1.50)	< 0.05	11.27	< 0.05	0.0	< 0.05
**Religion**							
Muslim	5	1.10 (1.07–1.12)	< 0.05	6.21	0.184	0.0	< 0.05

*Note:* POR, pooled odds ratio; CI, confidence interval; *Q*‐value = reflects study variability, *I* ^2^ (Tua‐square) = estimated variance of the observed effect sizes.

Children with age “6 to 11” months and “24 to 35” months showed the lowest and highest odds of stunting with 1.57 (95% CI: 1.06–2.08; *I* ^2^ = 95.41, *p *< 0.05, *n* = 13) and 3.31 (95% CI: 2.56–4.06; *I* ^2^ = 98.8, *p *< 0.05, *n* = 22) times higher likelihoods, respectively. Similarly, child age “36 to 47” months (POR: 3.16, 95% CI: 2.36–3.96; *I* ^2^ = 98.7, *p *< 0.05, *n* = 17), “12 to 23” months (POR: 2.67, 95% CI: 2.16–3.17; *I* ^2^ = 98.5, *p *< 0.05, *n* = 31), and child age “48 to 59” months (POR: 2.42, 95% CI: 1.72–3.13; *I* ^2^ = 98.9, *p *< 0.05, *n* = 18) were found higher odds of being stunted, respectively. According to child size at birth, this study found that small (POR: 1.97, 95% CI: 1.54–2.39; *I* ^2^ = 84.9, *p *< 0.05, *n* = 8) and average (POR: 1.07, 95% CI: 0.84–1.29; *I* ^2^ = 95.3, *p *= 0.06, *n* = 9) size child was higher stunted than large size child. Following low birth weight covered 1.95 (95% CI: 0.67–3.24; *I* ^2^ = 78.0, *p *= 0.22, *n* = 34) times higher children stunted. Children were 1.59 (95% CI: 1.29–1.89; *I* ^2^ = 98.65, *p *< 0.05, *n* = 12) times higher stunted if mothers' BMI were underweight. Mother's profession as a service holder affects children stunting 1.33 (95% CI: 1.19–1.47; *I* ^2^ = 0.0, *p *= 0.09, *n* = 5) times higher. In contrast, children were found 17% less stunted if the mother received “1 to 3” times ANC visits during her pregnancy period (POR: 0.83, 95% CI: 0.73–0.93; *I* ^2^ = 88.5, *p *= 0.22, *n* = 6). Similarly, children were found 34% (POR = 0.66, 95% CI: 0.37– 0.95; *I* ^2^ = 99.17, *p *= 0.37, *n* = 4) less stunted if children were breastfed exclusively. On the other hand, children were found to be 1.24 times more likely to be stunted (95% CI: 0.93–1.55; *I* ^2^ = 97.61, *p *< 0.05, *n* = 5) if a sibling of the child was older than 5. Furthermore, unimproved water conditions imply that children were 1.19 times more likely to be stunted (95% CI: 1.08–1.32; *I* ^2^ = 30.77, *p *< 0.05, *n* = 6) compared to improved water conditions. Likewise, unimproved sanitation conditions imply that children were 1.44 times more likely to be stunted (95% CI: 1.07–1.81; *I* ^2^ = 91.59, *p *= 0.35, *n* = 6). Moreover, childbirth orders “2–4” (POR: 1.49, 95% CI: 1.04–1.94; *I* ^2^ = 99.3, *p *< 0.05, *n* = 7) and “5 & greater” (POR: 1.33, 95% CI: 1.27–1.39; *I* ^2^ = 0.0, *p *= 0.12, *n* = 5) found higher odds of child stunting. Additionally, children suffering from diarrhea had a POR of 1.36 (95% CI: 1.19–1.52; *I* ^2^ = 54.89, *p *< 0.05, *n* = 10), indicating a higher likelihood of stunting. Finally, severe family food insecurity conditions cause higher child stunting (POR: 1.37, 95% CI: 1.24–1.50; *I* ^2^ = 0.0, *p *< 0.05, *n* = 5).

Table 3 presents prevailing risk factors associated with child wasting in developing countries from 2010 to 2022. Specifically, male children were found to be 1.31 times more likely to be wasted than females (95% CI: 1.19–1.43; *I* ^2^ = 24.18, *p *= 0.08, *n* = 7). In contrast, children from the poorest family were found 1.17 times more wasted (95% CI: 0.59–1.75; *I* ^2^ = 80.22, *p *= 0.24, *n* = 5), whereas poor, middle, rich, and rich family‐children were progressively less likely to be wasted (POR < 1). Moreover, children of illiterate mothers were found to have 1.75 times higher odds of being wasted (95% CI: 1.05–2.46; *I* ^2^ = 53.5, *p *< 0.05, *n* = 7), while children with educated mothers were found to be less wasted (POR < 1). In terms of age, children age group “6 to 11” months were found 1.10 (95% CI: 0.66–1.54; *I* ^2^ = 89.88, *p *= 0.19, *n* = 6) times higher wasted, conversely, child aged “12 to 23,” “24 to 35,” “36 to 47,” and “48 to 59” months found less (POR < 1) wasted, respectively. Furthermore, mother's BMI underweight implies children are 1.87 times higher wasted (95% CI: 1.24–2.51; *I* ^2^ = 63.86, *p *=0.09, *n* = 5), on the other hand, normal and overweight BMI imply children less likely to be wasted (POR < 1). Additionally, children who are suffering from diarrhea were 1.36 (95% CI: 1.15–1.56; *I* ^2^ = 18.61, *p *< 0.05, *n* = 7) times higher wasted. Similarly, the children who suffered from fever were 1.25 times higher wasted (95% CI: 1.15–1.35; *I* ^2^ = 19.65, *p *= 0.14, *n* = 5), respectively.

**TABLE 3:** Summary of meta‐analysis for associated factors of childhood wasting in developing countries, 2010–22.

Factors	No. of studies	POR (95% CI)	*p* value	Heterogeneity			Egger's test (*p*‐value)
				*Q*‐value	*p* value	*I* ^2^	
**Child Sex**							
Male	7	1.31 (1.19–1.43)	< 0.05	9.05	0.171	24.18	0.08
Female	9	0.84 (0.66–1.01)	< 0.05	51.38	< 0.05	95.42	0.16
**Residence**							
Rural	5	0.85 (0.79–0.90)	< 0.05	6.87	0.143	0.00	0.35
**Wealth index**							
Poorest	5	1.17 (0.59–1.75)	< 0.05	24.41	< 0.05	80.22	0.23
Poor	8	0.84 (0.78–0.89)	< 0.05	6.42	0.491	0.00	0.27
Middle	12	0.85 (0.74–0.97)	< 0.05	21.06	< 0.05	50.44	0.16
Rich	9	0.77 (0.72–0.83)	< 0.05	6.25	0.619	0.00	0.66
Richest	5	0.71 (0.52–0.89)	< 0.05	12.71	< 0.05	58.26	0.14
**Mother education**							
Illiterate	7	1.75 (1.05–2.46)	< 0.05	13.77	< 0.05	53.51	< 0.05
Primary	10	0.95 (0.89–1.01)	< 0.05	7.76	0.558	0.00	0.09
SSC	7	0.85 (0.65–1.05)	< 0.05	12.03	0.061	53.77	0.43
**Child age group**							
6 to 11	6	1.10 (0.66–1.54)	< 0.05	20.92	< 0.05	89.88	0.19
12 to 23	11	0.98 (0.78–1.18)	< 0.05	26.80	< 0.05	70.46	0.24
24 to 35	8	0.74 (0.29–1.19)	< 0.05	52.03	< 0.05	97.35	0.12
36 to 47	7	0.88 (0.12–1.64)	< 0.05	47.74	< 0.05	98.91	0.09
48 to 59	4	0.37 (0.28–0.47)	< 0.05	6.44	0.092	53.56	0.45
**Mother's BMI status**							
Underweight	5	1.87 (1.24–2.51)	< 0.05	8.84	0.065	63.86	0.09
Normal	6	0.85 (0.57–1.14)	< 0.05	20.43	< 0.05	87.13	0.08
Overweight	6	0.64 (0.46–0.82)	< 0.05	36.29	< 0.05	84.80	0.39
**Diarrhea**							
Yes	7	1.36 (1.15–1.56)	< 0.05	17.15	< 0.05	18.61	< 0.05
**Fever**							
Yes	5	1.25 (1.15–1.35)	< 0.05	6.10	0.192	19.65	0.14

*Note:* POR, pooled odds ratio; CI, confidence interval; *Q*‐value = reflects study variability, *I* ^2^ (Tua‐square) = estimated variance of the observed effect sizes.

Table 4 presents the meta‐summary statistics for associated risk factors of childhood underweight in developing countries from 2010 to 2022. Specifically, male children were (POR) 1.4 (95% CI: 1.13–1.68; *I* ^2^ = 83.60, *p *< 0.05, *n* = 11) times higher underweight than females. Similarly, children were 2.59 (95% CI: 0.93–4.27; *I* ^2^ = 83.5, *p *< 0.05, *n* = 6) times higher underweight when their mothers were illiterate, whereas mothers with primary and above level of education found less likely to be underweight (POR < 1). Based on the wealth index, children across all socioeconomic groups exhibited lower odds of being underweight (POR < 1). The PORs of children underweight were found to be 2.03 (95% CI: 0.99–3.07; *I* ^2^ = 18.01, *p *= 0.53, *n* = 4) when the child's father was illiterate. In terms of age, children aged “6 to 11” months were found to be 1.50 times more likely to be underweight (95% CI: 0.86–2.14; *I* ^2^ = 96.06, *p *< 0.05, *n* = 8), and children aged “48 to 59” months were found to be 1.81 times more likely to be underweight (95% CI: 1.31–2.30; *I* ^2^ = 96.98, *p *= 0.10, *n* = 11), respectively. Furthermore, children whose birth size was small found POR 2.09 (95% CI: 1.66–2.51; *I* ^2^ = 60.60, *p *= 0.47, *n* = 4), and for large birth size children were found less underweight (POR = 0.68; 95% CI: 0.49–0.86; *I* ^2^ = 92.66, *p *= 0.65, *n* = 4), respectively. Children were found 2.41 (95%CI: 1.75–3.06; *I* ^2^ = 84.0, *p *< 0.05, *n* = 8) times higher underweight when their mothers were underweight. In contrast, improved sanitation system covered less child underweight (POR = 0.79, 95%CI: 0.71–0.86; *I* ^2^ = 0.0, *p *= 0.41, *n* = 4). Additionally, children were found 1.36 times more underweight when their birth order was found “2–4” (POR: 1.36, 95% CI: 1.02–1.69; *I* ^2^ = 86.5, *p *= 0.17, *n* = 5). Children were found 1.36 (95% CI: 1.27–1.45; *I* ^2^ = 0.70, *p *< 0.05, *n* = 9) times higher underweight when they had suffered from diarrhea. Similarly, children found 1.43 (95% CI: 0.96–1.89; *I* ^2^ = 93.06, *p *= 0.12, *n* = 4) times higher underweight when they had suffered from anemia.

**TABLE 4:** Summary of meta‐analysis for associated factors of childhood underweight in developing countries, 2010–22.

Factors	No. of studies	POR (95% CI)	*p* value	Heterogeneity			Egger's test (*p*‐value)
				*Q*‐value	*p* value	*I* ^2^	
**Child Sex**							
Male	11	1.40 (1.13–1.68)	< 0.05	45.03	< 0.05	83.60	< 0.05
Female	6	0.89 (0.76–1.04)	< 0.05	34.18	< 0.05	89.50	0.94
Residence: Rural	8	0.96 (0.71–1.21)	< 0.05	121.77	< 0.05	97.20	< 0.05
**Wealth index**							
Poor	10	0.78 (0.66–0.89)	< 0.05	34.64	< 0.05	75.21	< 0.05
Middle	14	0.81 (0.66–0.96)	< 0.05	109.79	< 0.05	92.87	< 0.05
Rich	11	0.71 (0.53–0.89)	< 0.05	59.94	< 0.001	94.08	< 0.05
Richest	10	0.72 (0.43–1.01)	< 0.05	101.52	< 0.05	97.68	< 0.05
**Mother education**							
Illiterate	6	2.59 (0.93–4.27)	< 0.05	35.32	< 0.05	83.48	< 0.05
Primary	16	0.85 (0.80–0.90)	< 0.05	30.60	< 0.001	34.78	0.08
Less than SSC	4	0.81 (0.75–0.87)	< 0.05	4.49	0.213	5.05	0.18
SSC	9	0.73 (0.68–0.76)	< 0.05	17.07	< 0.05	0.00	< 0.05
SSC and above	7	0.70 (0.64–0.76)	< 0.05	5.45	0.487	0.00	0.12
HSC and above	4	0.46 (0.37–0.54)	< 0.05	6.59	0.086	53.13	0.91
**Father education**							
Illiterate	4	2.03 (0.99–3.07)	< 0.05	2.12	0.548	18.01	0.53
Primary	6	0.97 (0.90–1.04)	< 0.05	11.46	< 0.05	0.00	0.09
**Child age group (in months)**							
6 to 11	8	1.50 (0.86–2.14)	< 0.05	101.49	< 0.05	96.06	< 0.05
12 to 23	14	1.61 (1.09–2.13)	< 0.05	204.14	< 0.05	98.53	< 0.05
24 to 35	11	1.59 (1.18–2.00)	< 0.05	120.22	< 0.05	95.49	0.07
36 to 47	10	1.74 (1.25–2.22)	< 0.05	120.02	< 0.05	95.68	0.11
48 to 59	11	1.81 (1.31–2.30)	< 0.05	176.44	< 0.05	96.98	0.09
**Child size at birth**							
Small	4	2.09 (1.66–2.51)	< 0.05	7.39	0.060	60.60	0.47
Average	4	1.07 (0.83–1.31)	< 0.05	29.07	< 0.05	93.64	0.14
Large	4	0.68 (0.49–0.86)	< 0.05	58.52	< 0.05	92.66	0.65
**Mother's BMI status**							
Underweight	8	2.41 (1.75–3.06)	< 0.05	27.89	< 0.05	84.00	< 0.05
Normal	12	1.13 (0.78–1.49)	< 0.05	121.38	< 0.05	98.25	< 0.05
Overweight	11	0.55 (0.43–0.67)	< 0.05	201.34	< 0.05	92.32	0.73
**Sanitation status**							
Improved	4	0.79 (0.71–0.86)	< 0.05	1.79	0.616	0.00	0.41
**Birth order**							
2–4	5	1.36 (1.02–1.69)	< 0.05	12.48	< 0.05	86.49	0.17
Diarrhea	9	1.36 (1.27–1.45)	< 0.05	13.72	0.089	0.70	< 0.05
Anemia	4	1.43 (0.96–1.89)	< 0.05	24.20	< 0.05	93.06	0.12

*Note:* POR, pooled odds ratio; CI, confidence interval; *Q*‐value = reflects study variability, *I* ^2^ (Tua‐square) = estimated variance of the observed effect sizes.

As part of the sensitivity analysis, we excluded articles with extreme prevalence values. Specifically, for stunting, studies were excluded if the reported prevalence was below 10% (*n* = 5) or above 50% (*n* = 4). In the same manner, for wasting, studies with prevalence below 5% (*n* = 17) or above 30% (n = 2) were excluded. Likewise, for underweight, studies were excluded if the prevalence was below 10% (*n* = 11) or above 40% (*n* = 4). After excluding outliers in stunting, wasting, and underweight prevalence, we determined the pooled prevalence for stunting was 33% (95% CI: 31.3–34.7, *I* ^2^ = 99.7%, *p* < 0.05, *n* = 132), wasting 12.6% (95% CI: 11.4 – 13.7, *I* ^2^ = 99.1, *p *< 0.05, *n* = 83), and underweight 23.8% (95% CI: 21.9–25.6, *I* ^2^ = 99.6, *p *= 0.32, *n* = 78), respectively (see Table 5). Subgroup analysis demonstrated significant heterogeneity in the prevalence of childhood stunting across continents (*Q* = 493.6, *p* < 0.05) and over time (*Q* = 29.98, *p* < 0.05), reflecting considerable variation by continent and years from 2010 to 2022. Likewise, childhood wasting exhibited significant heterogeneity by continent (*Q* = 17.84, *p* < 0.05), but no significant variation was observed across years (*Q* = 8.8, *p* = 0.55), indicating geographic differences with temporal stability. Additionally, childhood underweight showed significant heterogeneity both by continent (*Q* = 50.97, *p* < 0.05) and by year (*Q* = 58.67, *p* < 0.05), underscoring marked variation across regions and years (2010–22). These outcomes closely align with the overall results, indicating the robustness of the findings.

**TABLE 5:** Pooled prevalence of childhood undernutrition in developing countries (2010–2022), including sensitivity analysis and subgroup analysis by continent and year.

Indicators	No. of studies	Prevalence (95% CI)	*p* value	Heterogeneity			Egger's test (*p*‐value)
				*Q*‐value	*p* value	*I* ^2^	
Stunting	132	33.0 (31.3–34.7)	< 0.05	35,162.8	< 0.05	99.7	< 0.05
**Subgroup analysis of Stunting for Continent**							
Africa	77	35.5 (33.5–37.5)	< 0.05	6133.5	< 0.05	99.0	
Asia	50	30.6 (27.9–33.3)	< 0.05	25,685.8	< 0.05	99.8	
North America	1	11.9 (10.2–13.6)	< 0.05	—	—	—	
Oceania	1	37.1 (33.9–40.3)	< 0.05	—	—	—	
South America	3	14.8 (13.1–16.5)	< 0.05	9.62	< 0.05	75.7	
**Subgroup analysis of Stunting for Year**							
2012	1	39.6 (34.4–44.8)	< 0.05	—	—	—	
2013	3	29.6 (14.3–45.0)	< 0.05	51.8	< 0.05	98.2	
2014	4	25.8 (15.8–35.8)	< 0.05	30.4	< 0.05	96.7	
2015	9	41.7 (38.3–45.1)	< 0.05	116.4	< 0.05	94.0	
2016	5	36.3 (30.6–42.0)	< 0.05	236.9	< 0.05	98.1	
2017	15	35.5 (31.3–39.7)	< 0.05	867.5	< 0.05	98.7	
2018	13	32.1 (26.7–37.5)	< 0.05	948.2	< 0.05	98.1	
2019	24	32.4 (28.4–36.4)	< 0.05	6319.9	< 0.05	99.6	
2020	22	31.1 (26.5–35.8)	< 0.05	6648.4	< 0.05	99.7	
2021	23	31.1 (26.6–35.6)	< 0.05	9023.9	< 0.05	99.7	
2022	13	33.9 (28.8–38.9)	< 0.05	2398.2	< 0.05	99.7	
Wasting	83	12.6 (11.4–13.7)	< 0.05	8424.9	< 0.05	99.1	< 0.05
**Subgroup analysis of Wasting for Continent**							
Africa	50	12.1 (10.5–13.7)	< 0.05	3601.8	< 0.05	99.1	
Asia	32	13.5 (11.9–15.1)	< 0.05	3617.9	< 0.05	98.9	
South America	1	7.0 (4.4–9.6)	< 0.05	—	—	—	
**Subgroup analysis of Stunting for Year**							
2012	1	11.3 (7.9–14.7)	< 0.05	—	—	—	
2013	3	16.8 (5.3–28.3)	< 0.05	42.2	< 0.05	97.9	
2014	2	12.3 (3.1–1.4)	< 0.05	7.6	< 0.05	86.8	
2015	6	14.4 (11.2–17.6)	< 0.05	37.4	< 0.05	89.7	
2016	3	10.7 (5.2–16.1)	< 0.05	167.4	< 0.05	99.0	
2017	9	15.4 (10.5–20.4)	< 0.05	953.5	< 0.05	99.4	
2018	3	8.5 (4.7–12.3)	< 0.05	23.4	< 0.05	91.1	
2019	17	11.4 (9.2–13.6)	< 0.05	927.3	< 0.05	99.1	
2020	14	12.6 (10.1–15.1)	< 0.05	1367.9	< 0.05	98.8	
2021	17	12.3 (9.8–14.8)	< 0.05	1733.1	< 0.05	99.4	
2022	8	12.5 (8.9–16.1)	< 0.05	1120.8	< 0.05	98.4	
Underweight	78	23.8 (21.9–25.6)	< 0.05	21,150.2	< 0.05	99.6	0.315
**Subgroup analysis of Underweight for Continent**							
Africa	46	21.0 (18.9–23.1)	< 0.05	5937.2	< 0.05	99.1	
Asia	31	28.1 (25.2–30.9)	< 0.05	7312.3	< 0.05	99.7	
South America	1	17.3 (16.2–18.4)	< 0.05	—	—	—	
**Subgroup analysis of Underweight for Year**							
2012	1	32.1 (27.1–37.1)	< 0.05	—	—	—	
2013	1	11.2 (7.3–37.1)	< 0.05	—	< 0.05	—	
2015	6	21.7 (14.5–28.9)	< 0.05	225.2	< 0.05	97.1	
2016	4	26.1 (17.2–35.1)	< 0.05	299.4	< 0.05	99.2	
2017	8	21.6 (17.3–25.9)	< 0.05	619.7	< 0.05	98.1	
2018	7	25.7 (19.7–31.6)	< 0.05	764.1	< 0.05	99.3	
2019	15	19.9 (15.9–23.8)	< 0.05	2581.9	< 0.05	99.4	
2020	15	23.5 (19.3–27.7)	< 0.05	7546.3	< 0.05	99.7	
2021	16	27.7 (24.1–31.3)	< 0.05	3023.8	< 0.05	99.3	
2022	5	26.4 (16.6–36.3)	< 0.05	713.9	< 0.05	99.5	

Finally, to more precisely assess the robustness of the study findings, subgroup analysis was conducted based on JBI quality scores. This analysis revealed a pooled prevalence of childhood stunting of 32.5% (95% CI: 29.6–35.4, *I* ^2^ = 99.8, *p *< 0.05, *n* = 35) in high‐quality studies, 33.0% (95% CI: 30.5–35.5, *I* ^2^ = 99.7, *p *< 0.05, *n* = 65) in medium‐quality studies, and 20.3% (95% CI: 0.5–40.1, *I* ^2^ = 99.5, *p *< 0.05, *n* = 2) in low‐quality studies (see Table 6). Similar for wasting, the pooled prevalence was found 11.6% (95% CI: 9.5–13.7, *I* ^2^= 99.7, *p *< 0.05, *n* = 35) in high‐quality studies, 11.6% (95% CI:9.8–13.3, *I* ^2^ = 99.5, *p *< 0.05, *n* = 65) in medium‐quality studies, and 9.4% (95% CI: 6.4–25.3, *I* ^2^ = 99.3, *p *< 0.05, *n* = 2) in low‐quality studies. Finally for underweight, pooled prevalence was found 23.1% (95% CI: 19.6–26.6, *I* ^2^ = 99.7, *p *< 0.05, *n* = 33) in high‐quality studies, 22.8% (95% CI: 19.9–25.7, *I* ^2^ = 99.8, *p *< 0.05, *n *= 58) in medium‐quality studies, and 9.4% (95% CI: 4.7–23.6, *I* ^2^ = 99.1, *p *< 0.05, *n *= 2) in low‐quality studies (see in Table 6).

**TABLE 6:** Pooled prevalence of childhood undernutrition in developing countries (2010–2022), subgroup analysis by JBI quality scores.

Indicators	No. of studies	Prevalence (95% CI)	*p* value	Heterogeneity		
				*Q*‐value	*p* value	*I* ^2^
**Subgroup analysis of Stunting for JBI quality scores**						
High Quality	35	32.5 (29.6–35.4)	< 0.05	41,211.9	< 0.05	99.8
Medium Quality	65	33.0 (30.5–35.5)	< 0.05	24,847.5	< 0.05	99.7
Low Quality	2	20.3 (0.5–40.1)	< 0.05	184.7	< 0.05	99.5
Overall: *Q* = 1.59, *p *= 0.452						
**Subgroup analysis of Wasting for JBI quality scores**						
High Quality		11.6 (9.5–13.7)	< 0.05	7727.6	< 0.05	99.5
Medium Quality		11.6 (9.8–13.3)	< 0.05	6234.9	< 0.05	99.5
Low Quality		9.4 (6.4–25.3)	< 0.05	135.1	< 0.05	99.3
Overall: *Q* = 0.07, *p *= 0.966						
**Subgroup analysis of Underweight for JBI quality scores**						
High Quality	33	23.1 (19.6–26.6)	< 0.05	9618.6	< 0.05	99.7
Medium Quality	58	22.8 (19.9–25.7)	< 0.05	45,217.8	< 0.05	99.8
Low Quality	2	9.4 (4.7–23.6)	< 0.05	109.1	< 0.05	99.1

*Note:* Overall: *Q* = 3.45, *p *= 0.179.

## 4 Discussions

Child undernutrition, characterized by stunting, wasting, and being underweight, represents fundamental aspects of child's nutritional status. This study conducted a systematic review and meta‐analysis to consolidate its findings. We estimated the pooled prevalence and identified the contributing factors to stunting, wasting, and underweight from 157 reviewed studies conducted in developing countries from 2010 to 2022.

Every year, children under the age of five are affected by undernutrition conditions like stunting, wasting, and low birth weight, particularly in developing countries [4]. Severe undernutrition is particularly common in developing countries, where the total pooled prevalence of stunting is significantly higher [37]. In Sub‐Saharan Africa, stunting impacted 41% of children [38]. Another study from 137 developing countries based on secondary data in 2010, found the average prevalence of stunting is 13.5% [39]. As per the 2022 World Bank Report, child stunting rates were recorded at 31.0% for Africa, and 30.10% for South‐East Asia [40]. A study for low‐ and middle‐income countries found the pooled prevalence of stunting is 29.1% [41]. While this study found 32.6% of stunting for under‐5 children, which is almost close to other findings. As recommended by additional research, the birth size and weight of a child have an impact on stunting as well [42]. Maternal undernutrition is intimately linked to the prenatal causes of children's stunted development [43]. Stunting risk can be decreased by exclusive breastfeeding and having lesser number of children [44], which represents our findings. Given the large concentration of growth‐promoting chemicals in breastfeeding, an effect on stunting is conceivable [19, 45]. Additionally, mothers who do not obtain high‐quality antenatal care (ANC) during their pregnancy are more likely to have stunted children [15]. This is due to the fact that ANC can offer a chance to detect health concerns that women already endure and to protect their offspring from health issues. On the other hand, maternal service does not appear to be lowering the rate of stunting [46], which is similar to our results.

According to the World Bank Report 2022, the prevalence of child wasting in South Asia is 14.8% [47]. Following three South Asian countries (Bangladesh, India, and Nepal), 11%–20% of children under 5 suffer from wasting [48]. In another study for South Asia claimed that 20% of children aged 0–59 months are affected by wasting [31]. Another study from 94 countries found the pooled prevalence of wasting to be 14% for children under 2 years old and 9% for children aged 2–4 years [49]. Moreover, other study reveals that in South Asia, the prevalence of wasting is 15% among children under 2 years old and 21% among those aged two to 4 years, whereas in lower‐middle‐income countries, it is 12% for children under 2 years old and 17% for those aged 2 to 4 years [49]. P. Ssentongo et al. prevailed for low‐ and middle‐income countries, covering 6.3% of wasting for children under 5 [41]. Based on 35 low‐ and middle‐income countries, Z. Li et al. found that 12.9% of children aged 12–59 months are wasted [50]. Additionally, our study found that 11.5% of wasted children. Even in some regions of Asia, the percentage of children affected by wasting has decreased across income levels, but the overall figure remains high [10]. O. R. Katoch et al. found that factors linked to child wasting include maternal body mass index (BMI) [41], age, wealth index, birth order [51] and interval, births occurring at home, and access to antenatal care visits [31]. These factors coincide with our findings. Additionally, fever‐stricken children are more likely to be wasted [23, 52], which is similar to our results. Moreover, variables including birth sequence, male gender, maternal lack of education, shorter maternal height, lack of access to improved water sources, and household poverty were associated with wasting across various nations [10]. All these indicators coincide with our findings.

According to the World Bank Report 2022, the prevalence of underweight in South Asia is 26% [53]. For low‐ and middle‐income countries, the prevalence of underweight is 13.7% [41] found by P. Ssentongo et al. Z. Li et al., prevailed for 35 LMICs that 27.5% of children aged 12–59 months are underweight [50]. In another study based on 31 sub‐Saharan African countries, approximately 21% of children were identified as underweight [54]. Our study found that 22.6% of children are underweight. Children afflicted with anemia are more likely to be underweight [55], which was also observed in our study. Moreover, undernutrition correlates with hygiene practices, such as access to improved water and sanitation [56]. Specifically, adequate sanitation practices can mitigate the risk of infections, particularly diarrhea. Factors like having access to an improved toilet rather than an unimproved one, and having onsite water access as opposed to collecting it externally, serve as protective measures against malnutrition in children under 5 years old [57]. Disparities in stunting and underweight tend to exacerbate with age, manifesting from 6 to 12 months and persisting until the child reaches 2 or 3 years of age [58]. S. Khandelwal et al. observed notably high rates of wasting and underweight prevalence in the Asian continent [59].

Comparing sexes, males consistently show a higher susceptibility to malnutrition compared to females in developing countries [38], which is aligned with our findings. This is attributed to boys being more prone to childhood afflictions such as lower respiratory infections, diarrheal illnesses, malaria, and preterm delivery, in contrast to girls [60]. Rural children face an increased risk of malnutrition [61, 62]. Similar way, age represents a significant risk factor when considering undernutrition [26, 63]. Furthermore, children who experience diarrhea often suffer from severe metabolic acidosis, severe dehydration, and inadequate circulation, making them more likely to be underweight, wasted, or stunted [64]. Z. Li et al. found that lack of maternal education, diarrhea, poor dietary diversity, low wealth index, short maternal stature [10, 12], unimproved sanitation, and lack of breastfeeding [65] are risk factors for undernutrition, respectively [50]. Research conducted in Sub‐Saharan Africa identified several key factors associated with childhood stunting, wasting, and underweight. These include maternal education levels, child's age, male gender, socioeconomic status, low birth weight, lack of access to improved water sources, maternal underweight (BMI < 18.5), small birth size, incidence of diarrhea, paternal education levels, and place of residence [12, 54]. The primary reason for the growing number of undernourished children in low‐income families is related to the parents' lower levels of education [66]. Therefore, parental education has long been recognized as a significant risk factor for undernutrition [15, 38]. Stunting, wasting, and malnutrition are among the poor nutritional outcomes associated with children of illiterate mothers [67]. Therefore, a higher socioeconomic status has a greater impact on preventing malnutrition in children under 5 [68]. Furthermore, several studies suggest a significant issue of malnutrition associated with food security [11, 38, 69]. The risk factors identified in this analysis for the overall three indicators have also been discovered in other research [51, 70, 71]. The developing countries, especially in Asia and Africa, include significant disparities in wealth and health for ethnic, religious, and socio‐economic groupings, which need to be addressed alongside to achieve the targets of SDG 2.2.

### 4.1 Study Strengths and Limitations

The comprehensive overview of under‐5 child undernutrition that our study presents includes stunting, wasting, and underweight, all of which are prevalent in developing countries. The findings of the pooled prevalence as well as the pooled odds of associated causes from the relevant research related to developing countries can serve as a general overview for any developing country worldwide to take the required actions in order to reduce undernutrition among children under five. The study also compares pooled prevalence across continents to address the undernutrition status. However, we acknowledge certain limitations of our study. Firstly, it did not allow for the examination of trends in undernutrition prevalence in developing countries. Second, the selection of English‐only studies. The study did not include research before 2010. Although overweight is a common form of malnutrition in developed countries, we are only considering three indicators that are common in developing countries. The retrieved related factors were extracted from prevalence‐based research. Thus, the study may be limited in the complete coverage of studies related to risk factors. Additionally, this study has limitations in controlling the potential for publication bias, as it includes only the available studies from the listed databases. Lastly, this study covered only four databases; articles can be added from more sources. In these circumstances, further study is required.

### 4.2 Recommendation

More than a quarter of children in developing countries are affected by stunting and around a quarter are underweight. The economic condition of a country or economic status of an individual family determines the occurrence of undernutrition. Investments in hygiene practice behaviors, access to safe drinking water, parental education, and sector‐wide public sector initiatives that take nutrition into account are desperately needed. Another important strategy to lessen the problem is to have pregnant mothers attend prenatal care frequently. Political will, community and cultural leaders' participation, effective communication, conferences, health education, and promotion should all be an effective part of solving the problem of undernutrition. In developing countries, health programmers and researchers would be inclined to investigate the disparities in child health. Additional investigations could encompass a variety of languages and expand the scope to include a broader range of electric databases than ours.

## 5 Conclusions

Due to the high morbidity rate among children under five, developing countries continue to have dubious public health conditions each year. Finding the prevalence and causes of undernutrition is the aim of this investigation. Also, provide an outline of the distribution of undernutrition among different continents. The study suggests the most common causes of all forms of undernutrition in developing countries are the child's sex, age, mother's BMI, parental education, socioeconomic position, rural‐urban environment, diarrhea, food security, and hygiene issues. It is imperative to prioritize childhood undernutrition and address the aforementioned contributing factors during interventions aimed at improving childhood nutrition in developing countries. Addressing these factors requires strengthening maternal health and nutrition programs, expanding access to quality education, and improving water, sanitation, and hygiene services. Interventions should be designed for long‐term sustainability.

## Author Contributions

Mossamet Kamrun Nesa contributed to conceptualization, database search, data screening, methodology, validation, drafting the original manuscript, and reviewing and editing. Md. Rashed Babu was involved in database search, data screening, formal analysis, methodology, software, visualization, drafting the original manuscript, and reviewing and editing. Sumaiya Tasnim contributed to data screening, methodology, drafting the original manuscript, and reviewing and editing. Md. Jamal Uddin was involved in conceptualization, database search, data screening, methodology, investigation, project administration, supervision, validation, and reviewing and editing. All authors have read and approved the final version of the manuscript. Md. Jamal Uddin had full access to all of the data in this study and takes complete responsibility for the integrity of the data and the accuracy of the data analysis.

## Ethics Statement

This systematic review and meta‐analysis did not require ethical approval as it did not involve direct interaction with patients, humans, or animals. However, the study received institutional approval from the Ethical Review Board of the International Center for Diarrheal Disease Research, Bangladesh (icddr,b) (RG‐01945).

## Conflicts of Interest

The authors declare no conflicts of interest.

## Transparency Statement

The lead author, Md. Jamal Uddin affirms that this manuscript is an honest, accurate, and transparent account of the study being reported; that no important aspects of the study have been omitted; and that any discrepancies from the study as planned (and, if relevant, registered) have been explained.

## Supporting information


**Appendix Figure S1:** Pooled prevalence of stunting in developing countries, 2010‐22, sub‐grouped by year and continent. **Appendix Figure S2:** Pooled prevalence of wasting in developing countries, 2010‐22, sub‐grouped by year and continent. **Appendix Figure S3:** Pooled prevalence of underweight in developing countries, 2010‐22, sub‐grouped by year and continent. **Appendix Figure S4:** Funnel plot for prevalence of childhood stunting in developing countries. **Appendix Figure S5:** Funnel plot for prevalence of childhood wasting in developing countries. **Appendix Figure S6:** Funnel plot for prevalence of childhood underweight in developing countries.

**Appendix Table S1:** PRISMA 2020 Checklist for abstract. **Appendix Table S2:** PRISMA 2020 checklist for manuscript. **Appendix Table S3:** Inclusion and exclusion criteria. **Appendix Table S4:** Extracted information of eligible studies. **Appendix Table S5:** Quality assessment extracted from articles.

Supplementary Information

## Data Availability

Data described in the manuscript, code book, and analytic code will be made available upon request to corresponding author (jamal-sta@sust.edu).
