# Supplementary material for: Assessment of Undernutrition Among Under 5 Children in Developing Countries: A Systematic Review and Meta‐Analysis
Source: Health Sci Rep. 2026 Mar 16;9(3):e72087. doi: 10.1002/hsr2.72087 (PMC13098099; doi:10.1002/hsr2.72087)
Supplement: Supplementary file 1 — Appendix Figure S1: Pooled prevalence of stunting in developing countries, 2010‐22, sub‐grouped by year and continent. Appendix Figure S2: Pooled prevalence of wasting in developing countries, 2010‐22, sub‐grouped by year and continent. Appendix Figure S3: Pooled prevalence of underweight in developing countries, 2010‐22, sub‐grouped by year and continent. Appendix Figure S4: Funnel plot for prevalence of childhood stunting in developing countries. Appendix Figure S5: Funnel plot for prevalence of childhood wasting in developing countries. Appendix Figure S6: Funnel plot for prevalence of childhood underweight in developing countries. [file HSR2-9-e72087-s001.docx]

**Search keywords:**

(“prevalence” or “percentage” or “incidence”) and (“stunting” or “wasting” or “underweight” or “malnutrition” or “undernutrition” or “nutritional status” or “protein-energy malnutrition” or “child nutrition disorder” or “infant nutrition disorder”) AND (“child” or “under-five” or “under-five-children” or “preschool” or “pediatric” or “infant”).

Appendix **Table S3.** Inclusion and exclusion criteria

| **Criteria** | **Inclusion criteria** | **Exclusion criteria** |
| --- | --- | --- |
| Age | Child under five | Child greater than five, Adolescent, and adult |
| Outcome | - Prevalence or percentage or incidence of undernutrition. |  |
| Study design | - Observational studies - Surveys - Cross sectional studies | - Randomized controlled trials / clinical trials - Intervention studies - Facility based/special population studies - Editorials, letters, and commentaries - Case studies, reports, or case series - Theses and dissertations - Narrative literature reviews - Guidelines - Review article - Cohort study. - Longitudinal Study |
| Language | English | Non-English |
| Region/Country | LMICs, Developing Countries | Developed countries |
|  |  | - Not conducted in humans - Not community-based studies |
| Duration of time | 01 January 2010 to 31 December, 2022 | Except for this range. |

Appendix **Table S4:** Extracted information of eligible studies.

| **Author name** | **Publication Year** | **Continent** | **Country** | **Sample size** | **Data source** | **Stunting** | **Wasting** | **Underweight** |
| --- | --- | --- | --- | --- | --- | --- | --- | --- |
| Akram et al. | 2018 | Asia | Bangladesh | 863 | SD | 36.30 | **-** | **-** |
| Ranathunga et al. | 2021 | Asia | Sri Lanka | 262 | PD | 10.40 | 17.10 | 15.40 |
| Geda et al. | 2021 | Africa | Ethiopia | 9218 | SD | 38.00 | 9.40 | 25.20 |
| Amare et al. | 2013 | Africa | West Africa | 872 | PD | 21.80 | 9.40 | 3.70 |
| Huda et al. | 2020 | Africa | Uganda | 4765 | SD | 27.00 | 4.00 | 17.00 |
| Chavez-Zarate et al. | 2018 | South America | Peru | 4637 | SD | 15.90 | **-** | 17.30 |
| Renzaho et al. | 2017 | Africa | Ghana | 7550 | SD | 27.50 | 7.70 | 17.30 |
| Widyaningsih et al. | 2022 | Asia | Indonesia | 3887 | SD | 28.71 | **-** | 38.81 |
| Kang et al. | 2018 | Asia | Bhutan | 1506 | SD | 21.20 | 2.60 | 7.40 |
| Roba et al. | 2020 | Africa | Ethiopia | 1091 | PD | 53.90 | 16.80 | 36.90 |
| Aiga et al. | 2020 | Africa | Mozambique | 1498 | PD | 46.20 | 7.10 | 20.00 |
| Banerjee et al. | 2020 | Asia | India | 225002 | SD | 52.19 | **-** | **-** |
| Sarma et al. | 2017 | Asia | Bangladesh | 7647 | SD | 41.00 | **-** | **-** |
| Alaofe et al. | 2019 | Africa | Benin | 426 | PD | 37.60 | 10.10 | 22.80 |
| Blankenship et al. | 2020 | Oceania | Marshall Island | 881 | SD | 37.10 | 4.00 | **-** |
| Wasihun et al. | 2018 | Africa | Ethiopia | 610 | PD | 36.10 | 5.40 | 7.90 |
| Ademas et al. | 2021 | Africa | Ethiopia | 630 | PD | 35.60 | **-** | **-** |
| Mya et al. | 2019 | Asia | Myanmar | 1222 | SD | 20.00 | **-** | **-** |
| Yao et al. | 2015 | Africa | Malawi | 196 | PD | 39.00 | 2.00 | 13.00 |
| Kumar et al. | 2016 | Africa | Ethiopia | 541 | PD | 41.40 | 6.30 | 28.70 |
| Shah et al. | 2015 | Asia | Sri Lanka | 340 | PD | 7.10 | 21.20 | 16.90 |
| Ihejirika et al. | 2019 | Africa | Cameroon | 649 | PD | 31.30 | 6.30 | 13.10 |
| Pileggi et al. | 2017 | Africa | Bangui | 414 | PD | 36.00 | 4.00 | **-** |
| Iddrisu et al. | 2021 | Asia | Vietnam | 327 | PD | 23.50 | 6.70 | 7.60 |
| Hagos et al. | 2017 | Africa | Ethiopia | 2371 | PD | 43.70 | **-** | **-** |
| Rasheed et al. | 2020 | Asia | Maldives | 714 | PD | 32.40 | 16.30 | 24.60 |
| Gani et al. | 2020 | Africa | Ghana | 581 | PD | 33.20 | 14.10 | 27.00 |
| Sharma et al. | 2018 | Asia | Yemen | 1292 | PD | 38.50 | 39.90 | 55.10 |
| Abbas et al. | 2021 | Asia | Pakistan | 7781 | SD | **-** | 21.00 | **-** |
| Kassie et al. | 2019 | Africa | Ethiopia | 8768 | SD | 36.34 | 12.09 | 24.80 |
| Humbwavali et al. | 2012 | Asia | China | 336 | PD | 39.58 | 11.31 | 32.14 |
| Mshida et al. | 2018 | Africa | Tanzania | 310 | PD | 31.60 | 4.50 | 15.50 |
| Sunguya et al. | 2019 | Africa | Ethiopia | 394 | PD | **-** | 7.20 | **-** |
| Mekonen et al. | 2019 | Africa | Ethiopia | 616 | PD | 44.40 | **-** | **-** |
| Sartoriuset al. | 2015 | Africa | Uganda | 104 | PD | 38.50 | 16.50 | 13.50 |
| Ghosh et al. | 2021 | Asia | Bangladesh | 7173 | SD | 12.30 | 3.20 | 3.00 |
| Haque et al. | 2021 | Asia | Bangladesh | 5069 | PD | 44.40 | 10.50 | 31.90 |
| Ali et al. | 2019 | Asia | Bangladesh | 6468 | PD | 36.80 | 18.20 | 37.70 |
| Angela et al. | 2020 | Asia | Nepal | 5479 | SD | 34.10 | 13.70 | **-** |
| Kang et al. | 2019 | Asia | Myanmar | 4550 | SD | 29.00 | 7.30 | 19.20 |
| Acquah et al. | 2019 | Africa | Ghana | 2720 | SD | **-** | **-** | 11.00 |
| Boulom et al. | 2022 | Asia | Laos | 264 | SD | 54.90 | 17.00 | 46.20 |
| Susiloretni et al. | 2021 | Asia | Indonesia | 46315 | SD | 20.50 | **-** | **-** |
| Flynn et al. | 2021 | Asia | Indonesia | 408 | PD | 53.90 | **-** | 29.17 |
| Geberselassie | 2018 | Africa | Ethiopia | 1287 | SD | 49.40 | **-** | **-** |
| Geresomo | 2017 | Asia | India | 306 | PD | 31.20 | **-** | **-** |
| Al-Zangabila et al. | 2021 | Asia | Yemen | 13624 | SD | 47.00 | 16.00 | 39.00 |
| Kebede et al. | 2021 | Africa | Ethiopia | 974 | SD | 46.30 | 9.80 | 28.40 |
| Murarkar et al. | 2020 | Asia | India | 3671 | PD | 45.90 | 17.10 | 35.40 |
| Zewdie et al. | 2013 | Africa | Ethiopia | 249 | PD | 45.80 | 28.90 | 11.20 |
| Dereje et al. | 2020 | Africa | Ethiopia | 160 | PD | 45.00 | **-** | **-** |
| Yirga et al. | 2019 | Asia | Pakistan | 3071 | SD | 44.40 | 10.70 | 29.40 |
| Mgongo et al. | 2017 | Africa | Tanzania | 1870 | PD | 41.90 | 24.70 | 46.00 |
| Rahman et al. | 2016 | Asia | Bangladesh | 7530 | SD | 41.10 | 15.80 | 36.20 |
| Mbwana et al. | 2017 | Africa | Tanzania | 120 | PD | 41.00 | **-** | **-** |
| Makori et al. | 2018 | Africa | Tanzania | 394 | SD | 40.40 | **-** | **-** |
| Djuardi et al. | 2021 | Asia | Indonesia | 393 | PD | 40.20 | 17.10 | 33.10 |
| Rahman | 2015 | Asia | Bangladesh | 6506 | SD | 40.00 | 16.00 | 35.00 |
| Busse et al. | 2018 | Africa | Ethiopia | 292 | PD | 26.70 | **-** | 34.60 |
| Sk R et al. | 2021 | Asia | India | 731 | PD | 40.00 | **-** | **-** |
| Gebreayohanes et al. | 2022 | Africa | Ethiopia | 554 | PD | 39.50 | **-** | **-** |
| Mahmudiono et al. | 2017 | Africa | Madagascar | 736 | PD | 39.40 | **-** | **-** |
| Kasaye et al. | 2019 | Africa | Ethiopia | 9494 | SD | 38.30 | 10.10 | 23.30 |
| Paul et al. | 2022 | Asia | India | 41158 | SD | 38.00 | 21.00 | 35.00 |
| Naz et al. | 2020 | Asia | Pakistan | 3575 | SD | 38.00 | 8.00 | 23.00 |
| Ndagijimana et al. | 2022 | Africa | Rwanda | 3813 | SD | 37.70 | **-** | **-** |
| Mengesha et al. | 2021 | Africa | Ethiopia | 600 | PD | 37.70 | **-** | **-** |
| Mann et al. | 2021 | Africa | Nigeria | 12996 | SD | 37.00 | 6.90 | 22.00 |
| Asgary et al. | 2015 | Africa | Madagascar | 313 | SD | 36.20 | 10.30 | **-** |
| Rahman et al. | 2021 | Asia | Bangladesh | 7079 | SD | 35.40 | 15.40 | 32.80 |
| McKenna et all. | 2019 | Africa | Congo | 3721 | SD | 35.20 | 9.20 | **-** |
| Joe et al. | 2019 | Asia | India | 12162 | SD | 28.00 | 12.80 | 24.30 |
| Hossain et al. | 2020 | Asia | Bangladesh | 10875 | SD | 24.67 | 9.75 | 20.57 |
| Mugode et al. | 2018 | Africa | Zambia | 295 | PD | 40.00 | **-** | **-** |
| Bedatu et al. | 2020 | Africa | Ethiopia | 408 | PD | 21.80 | 17.20 | 15.20 |
| Sartika et al. | 2021 | Asia | Indonesia | 559 | SD | 28.40 | 14.40 | 25.50 |
| Rahman et al. | 2021 | Asia | Bangladesh | 7738 | SD | 19.20 | 7.00 | 16.40 |
| Boah et al. | 2019 | Africa | Ghana | 2720 | SD | 18.40 | 5.30 | 10.40 |
| Campbell et al. | 2017 | Asia | Bhutan | 441 | SD |  | 5.30 | 10.40 |
| Christian et al. | 2020 | Africa | Ghana |  | SD | 13.30 | **-** | **-** |
| Islam et al. | 2019 | Asia | Bangladesh | 6965 | SD | 12.60 | 3.20 | 3.00 |
| Gamecha et al. | 2017 | Africa | Ethiopia | 595 | PD | 45.70 | 14.10 | 20.50 |
| Yeshaneh et al. | 2022 | Africa | Ethiopia | 2169 | PD |  | 14.70 | **-** |
| Salman et al. | 2020 | Africa | Nigeria | 12812 | SD | 43.30 | 18.70 | 29.00 |
| Obeyelu et al. | 2021 | Africa | Nigeria | 17199 | SD | 38.90 | 10.60 | 38.10 |
| Sethy et al. | 2014 | Asia | India | 144 | SD | 42.40 | 17.40 | 44.40 |
| Kiarie et al. | 2016 | Africa | Kenya | 204 | PD | 41.00 | 3.00 | 14.00 |
| Rajpal et al. | 2020 | Asia | India | 225002 | SD | 38.47 | **-** | 35.73 |
| Humbwavali et al. | 2014 | Africa | Angola | 749 | PD | 18.80 | **-** | **-** |
| Elnadif | 2020 | Africa | Sudan | 768 | PD | 13.50 | 17.60 | 16.70 |
| Jude et al. | 2019 | Africa | Nigeria | 782 | PD | 3.50 | 2.40 | **-** |
| Manyong et al. | 2021 | Africa | Nigeria | 1642 | PD | **-** | **-** | 19.70 |
| Haq et al. | 2022 | Asia | Pakistan | 10080 | SD | 29.71 | **-** | **-** |
| Yani et al. | 2019 | Africa | Malawi | 5786 | SD | 39.00 | 11.20 | 10.00 |
| Masibo et al. | 2022 | Africa | Ethiopia | 612 | PD | **-** | 14.10 | **-** |
| Baye et al. | 2022 | Africa | Lesotho | 3112 | SD | 43.00 | **-** | **-** |
| Anato | 2021 | North America | Maxico | 1338 | SD | 11.90 | **-** | **-** |
| Zembe-Mkabile et al. | 2019 | Africa | Angola | 749 | PD | 32.00 |  | 15.10 |
| Parra et al. | 2018 | Asia | Azerbaijan | 1455 | PD | 18.00 | 3.10 | **-** |
| Thompson et al. | 2022 | Africa | Somalia | 1947 | SD | 17.20 | 11.00 | **-** |
| Lassi et al. | 2020 | South America | Brazil | 372 | PD | 15.10 | 7.00 | **-** |
| Miller et al. | 2022 | Africa | Ethiopia | 903 | PD | 39.50 | 3.90 | **-** |
| Gordon et al. | 2020 | Asia | Pakistan | 25067 | SD | 27.00 | **-** | **-** |
| Garemo et al. | 2019 | Africa | Rwanda | 138 | PD | 42.00 | **-** | **-** |
| Belizario et al. | 2020 | Asia | Mongolia | 938 | SD | 6.30 | **-** | **-** |
| Elduma | 2015 | Africa | Tanzania | 678 | PD | 49.70 | **-** | **-** |
| Denney et al. | 2019 | Africa | Ethiopia | 394 | PD | 49.20 | **-** | **-** |
| Tariku et al. | 2020 | Africa | Ethiopia | 656 | PD | 47.90 | **-** | **-** |
| Danaei et al. | 2015 | Africa | Ethiopia | 796 | PD | 47.60 | 13.40 | 29.20 |
| Vieira et al. | 2015 | Africa | Ethiopia | 791 | PD | 45.80 | 10.70 | 21.00 |
| Grajeda et al. | 2017 | Africa | Ethiopia | 508 | PD | 45.60 | 14.60 | 26.30 |
| Tariq et al. | 2022 | Africa | Ethiopia | 354 | PD | 42.70 | 9.90 | 27.70 |
| Otsuka et al. | 2015 | Africa | Rwanda | 1882 | PD | 41.30 | **-** | **-** |
| Engidaye et al. | 2019 | Africa | Ethiopia | 841 | PD | 40.00 | 2.00 | 19.80 |
| Savanur et al. | 2022 | Africa | Ethiopia | 8855 | SD | 38.70 | **-** | **-** |
| Wali et al. | 2022 | Asia | India | 259002 | SD | 38.70 | **-** | **-** |
| Ferreira et al. | 2019 | Africa | Ethiopia | 9495 | SD | 38.30 | 10.10 | 23.30 |
| Perdono et al. | 2021 | Africa | Nigeria | 11314 | SD | 36.20 | 6.70 | 21.40 |
| Asoba et al. | 2020 | Asia | Nepal | 5060 | SD | 35.80 | **-** | 27.10 |
| Tafese et al. | 2015 | Africa | Tanzania | 7324 | SD | 35.50 | **-** | **-** |
| Ekholuenetale et al. | 2022 | Africa | Ethiopia | 626 | PD | 34.10 | 6.90 | 11.90 |
| Demmer et al. | 2019 | Africa | Tanzania | 2960 | SD | 31.00 | 14.00 | 6.00 |
| Di Cesare et al. | 2016 | Asia | Bangladesh | 10291 | PD | 30.90 | 9.70 | 24.90 |
| Richard et al. | 2017 | Africa | Kenya | 380 | PD | 29.20 | **-** | 20.80 |
| Oliphant et al. | 2019 | Africa | Ethiopia | 1768 | PD | 28.40 | 10.00 | 13.50 |
| Choudhury et al. | 2016 | Asia | Indonesia | 1366 | PD | 28.40 | **-** | **-** |
| yang et al. | 2018 | Asia | Pakistan | 984 | SD | 28.30 | 12.10 | 27.90 |
| Ayana et al. | 2020 | Africa | Kenya | 7830 | SD | 26.30 | 5.10 | 12.80 |
| Yim et al. | 2021 | Africa | Ethiopia | 316 | PD | 22.20 | 9.20 | 9.20 |
| Tonguet-Papucci et al. | 2021 | Africa | Africa | 774 | PD | 31.00 | 24.20 | **-** |
| Tasnim et al. | 2014 | Africa | Uganda | 442 | PD | 21.00 | 8.00 | **-** |
| Gatica-Dominguez et al. | 2017 | Asia | Palestine | 357 | PD | 19.60 | **-** | **-** |
| Alebel et al. | 2022 | Africa | Gambia | 2399 | SD | 13.50 | 5.90 | 18.70 |
| Gebremedhin | 2019 | Africa | Ethiopia | 17133 | SD | **-** | **-** | 31.67 |
| Gausman et al. | 2017 | Africa | Ethiopia | 398 | PD | **-** | 9.00 | 27.60 |
| Rivadeneira et al. | 2018 | Africa | Ethiopia | 645 | PD | 36.60 | 8.10 | 19.50 |
| Silveira et al. | 2019 | Africa | Burkino Faso | 956 | SD | **-** | 25.00 | **-** |
| Lagares et al. | 2019 | Asia | India | 18898 | SD | **-** | 14.80 | **-** |
| Wali et al. | 2022 | Asia | India | 259002 | SD | **-** | **-** | **-** |
| Fenske et al. | 2019 | Africa | Ethiopia | 8855 | SD | **-** | **-** | **-** |
| Ramos et al. | 2017 | Asia | Nepal | 4853 | PD | 37.90 | 17.80 | **--** |
| Dorsey et al. | 2018 | Asia | Pakistan | 24042 | SD | **-** | **-** | 33.32 |
| Hanna | 2020 | Africa | Ethiopia | 5467 | PD | 19.60 | 3.20 | **-** |
| Mutisya et al. | 2017 | Africa | Nigeria | 287 | PD | 23.70 | 28.90 | 21.30 |
| Ginsburg et al. | 2018 | Asia | Bangladesh | 8855 | SD | 38.40 | **-** | **-** |
| Rajoo et al. | 2021 | Asia | India | 360 | PD | 31.10 | 36.40 | 35.30 |
| Sanchez et al. | 2017 | Africa | Nigeria | 24529 | SD | 29.00 | **-** | **-** |
| Scharf et al. | 2013 | Africa | Angola | 744 | PD | 22.00 | 13.00 | 7.00 |
| Kansu et al. | 2019 | Asia | India | 13548 | SD | 17.10 | **-** | **-** |
| Akseer et al. | 2019 | South America | Peru | 4452 | SD | 13.60 | **-** | **-** |
| Prasad et al. | 2020 | Asia | Oman | 2945 | PD | 11.40 | 9.30 | 11.20 |
| Saeidlou et al. | 2014 | Asia | Iran | 2525 | PD | 7.30 | 1.40 | 2.30 |
| Saeidlou et al. | 2014 | Asia | Iran | 902 | PD | 23.10 | 4.90 | 8.10 |
| Vonaesch et al. | 2021 | Africa | Tanzania | 831 | PD | 1.60 | 4.20 | 29.80 |
| Dipasquale et al. | 2017 | Africa | Nigeria | 24529 | PD | **-** | 18.00 | 29.00 |
| Madiba et al. | 2021 | Asia | Nepal | 3169 | PD | 28.00 | 8.50 | **-** |
| Hartono et al. | 2020 | Asia | Indonesia | 300 | PD | 22.70 | **-** | **-** |

SD: Secondary data; PD: Primary data

Appendix **Table S5:** Quality assessment extracted from articles**.**

| **Author’s name** | **Publication**  **Year** | **JBI Score** | **Quality** | Q1 | Q2 | Q3 | Q4 | Q5 | Q6 | Q7 | Q8 |
| --- | --- | --- | --- | --- | --- | --- | --- | --- | --- | --- | --- |
| Akram et al. [73] | 2018 | 7 | High quality | 1 | 1 | 1 | 1 | 1 | 3 | 1 | 1 |
| Ranathunga et al. [74] | 2021 | 6 | Medium quality | 1 | 1 | 1 | 1 | 0 | 0 | 1 | 1 |
| Geda et al. [75] | 2021 | 7 | High quality | 1 | 1 | 1 | 3 | 1 | 1 | 1 | 1 |
| Amare et al. [76] | 2013 | 6 | Medium quality | 1 | 1 | 1 | 1 | 0 | 0 | 1 | 1 |
| Huda et al. [78] | 2020 | 8 | High quality | 1 | 1 | 1 | 1 | 1 | 1 | 1 | 1 |
| Chavez-Zarate et al. [79] | 2018 | 7 | High quality | 1 | 1 | 1 | 1 | 1 | 3 | 1 | 1 |
| Renzaho et al.[80] | 2017 | 8 | High quality | 1 | 1 | 1 | 1 | 1 | 1 | 1 | 1 |
| Widyaningsih et al. [81] | 2022 | 7 | High quality | 1 | 1 | 1 | 1 | 1 | 3 | 1 | 1 |
| Kang et al. [82] | 2018 | 7 | High quality | 1 | 1 | 1 | 1 | 1 | 3 | 1 | 1 |
| Roba et al. [85] | 2020 | 6 | Medium quality | 1 | 1 | 1 | 1 | 0 | 9 | 1 | 1 |
| Aiga et al. [86] | 2020 | 5 | Medium quality | 1 | 1 | 3 | 1 | 0 | 9 | 1 | 1 |
| Banerjee et al. [87] | 2020 | 8 | High quality | 1 | 1 | 1 | 1 | 1 | 1 | 1 | 1 |
| Sarma et al. [88] | 2017 | 6 | Medium quality | 1 | 1 | 1 | 1 | 0 | 9 | 1 | 1 |
| Alaofe et al. [89] | 2019 | 6 | Medium quality | 1 | 1 | 1 | 1 | 0 | 9 | 1 | 1 |
| Blankenship et al. [90] | 2020 | 8 | High quality | 1 | 1 | 1 | 1 | 1 | 1 | 1 | 1 |
| Wasihun et al. [91] | 2018 | 6 | Medium quality | 1 | 1 | 1 | 1 | 0 | 9 | 1 | 1 |
| Ademas et al. [92] | 2021 | 6 | Medium quality | 1 | 1 | 1 | 1 | 0 | 9 | 1 | 1 |
| Mya et al. [94] | 2019 | 6 | Medium quality | 1 | 1 | 1 | 1 | 0 | 9 | 1 | 1 |
| Yao et al. [96] | 2015 | 8 | High quality | 1 | 1 | 1 | 1 | 1 | 1 | 1 | 1 |
| Kumar et al. [97] | 2016 | 6 | Medium quality | 1 | 1 | 1 | 1 | 0 | 9 | 1 | 1 |
| Shah et al. [99] | 2015 | 6 | Medium quality | 1 | 1 | 1 | 1 | 0 | 9 | 1 | 1 |
| Ihejirika et al. [100] | 2019 | 8 | High quality | 1 | 1 | 1 | 1 | 1 | 1 | 1 | 1 |
| Pileggi et al. [101] | 2017 | 6 | Medium quality | 1 | 1 | 1 | 1 | 0 | 9 | 1 | 1 |
| Iddrisu et al. [102] | 2021 | 6 | Medium quality | 1 | 1 | 1 | 1 | 0 | 9 | 1 | 1 |
| Hagos et al. [103] | 2017 | 6 | Medium quality | 1 | 1 | 1 | 1 | 0 | 9 | 1 | 1 |
| Rasheed et al. [104] | 2020 | 6 | Medium quality | 1 | 1 | 1 | 1 | 0 | 9 | 1 | 1 |
| Gani et al. [105] | 2020 | 6 | Medium quality | 1 | 1 | 1 | 1 | 0 | 9 | 1 | 1 |
| Sharma et al. [106] | 2018 | 6 | Medium quality | 1 | 1 | 1 | 1 | 0 | 9 | 1 | 1 |
| Abbas et al. [107] | 2021 | 5 | Medium quality | 3 | 1 | 1 | 1 | 0 | 9 | 1 | 1 |
| Kassie et al. [108] | 2019 | 6 | Medium quality | 1 | 1 | 1 | 1 | 0 | 9 | 1 | 1 |
| Humbwavali et al. [109] | 2012 | 8 | High quality | 1 | 1 | 1 | 1 | 1 | 1 | 1 | 1 |
| Mshida et al. [110] | 2018 | 6 | Medium quality | 1 | 1 | 1 | 1 | 0 | 9 | 1 | 1 |
| Sunguya et al. [111] | 2019 | 6 | Medium quality | 1 | 1 | 1 | 1 | 0 | 9 | 1 | 1 |
| Mekonen et al. [112] | 2019 | 6 | Medium quality | 1 | 1 | 1 | 1 | 0 | 9 | 1 | 1 |
| Sartoriuset al. [113] | 2015 | 6 | Medium quality | 1 | 1 | 1 | 1 | 0 | 9 | 1 | 1 |
| Ghosh et al. [114] | 2021 | 6 | Medium quality | 1 | 1 | 1 | 1 | 0 | 9 | 1 | 1 |
| Haque et al. [115] | 2021 | 6 | Medium quality | 1 | 1 | 1 | 1 | 0 | 9 | 1 | 1 |
| Ali et al. [116] | 2019 | 6 | Medium quality | 1 | 1 | 1 | 1 | 0 | 9 | 1 | 1 |
| Angela et al. [117] | 2020 | 6 | Medium quality | 1 | 1 | 1 | 1 | 0 | 9 | 1 | 1 |
| Kang et al. [118] | 2019 | 6 | Medium quality | 1 | 1 | 1 | 1 | 0 | 9 | 1 | 1 |
| Acquah et al. [120] | 2019 | 6 | Medium quality | 1 | 1 | 1 | 1 | 0 | 9 | 1 | 1 |
| Boulom et al. [121] | 2022 | 6 | Medium quality | 1 | 1 | 1 | 1 | 0 | 9 | 1 | 1 |
| Susiloretni et al. [122] | 2021 | 6 | Medium quality | 1 | 1 | 1 | 1 | 0 | 9 | 1 | 1 |
| Flynn et al. [123] | 2021 | 6 | Medium quality | 1 | 1 | 1 | 1 | 0 | 9 | 1 | 1 |
| Geberselassie [124] | 2018 | 6 | Medium quality | 1 | 1 | 1 | 1 | 0 | 9 | 1 | 1 |
| Geresomo [125] | 2017 | 6 | Medium quality | 1 | 1 | 1 | 1 | 0 | 9 | 1 | 1 |
| Al-Zangabila et al. [126] | 2021 | 6 | Medium quality | 1 | 1 | 1 | 1 | 0 | 9 | 1 | 1 |
| Kebede et al. [127] | 2021 | 5 | Medium quality | 3 | 1 | 1 | 1 | 0 | 9 | 1 | 1 |
| Murarkar et al. [128] | 2020 | 8 | High quality | 1 | 1 | 1 | 1 | 1 | 1 | 1 | 1 |
| Zewdie et al. [129] | 2013 | 7 | High quality | 1 | 1 | 1 | 1 | 1 | 3 | 1 | 1 |
| Dereje et al. [130] | 2020 | 8 | High quality | 1 | 1 | 1 | 1 | 1 | 1 | 1 | 1 |
| Yirga et al. [131] | 2019 | 6 | Medium quality | 1 | 1 | 1 | 1 | 0 | 9 | 1 | 1 |
| Mgongo et al. [132] | 2017 | 6 | Medium quality | 1 | 1 | 1 | 1 | 0 | 9 | 1 | 1 |
| Rahman et al. [133] | 2016 | 8 | High quality | 1 | 1 | 1 | 1 | 1 | 1 | 1 | 1 |
| Mbwana et al. [134] | 2017 | 6 | Medium quality | 1 | 1 | 1 | 1 | 0 | 9 | 1 | 1 |
| Makori et al. [135] | 2018 | 4 | Low quality | 3 | 1 | 1 | 3 | 1 | 0 | 9 | 1 |
| Djuardi et al. [136] | 2021 | 7 | High quality | 1 | 1 | 1 | 1 | 1 | 3 | 1 | 1 |
| Rahman [137] | 2015 | 7 | High quality | 1 | 1 | 1 | 1 | 1 | 3 | 1 | 1 |
| Busse et al. [138] | 2018 | 8 | High quality | 1 | 1 | 1 | 1 | 1 | 1 | 1 | 1 |
| Sk R et al. [139] | 2021 | 8 | High quality | 1 | 1 | 1 | 1 | 1 | 1 | 1 | 1 |
| Gebreayohanes et al. [140] | 2022 | 6 | Medium quality | 1 | 1 | 1 | 1 | 0 | 9 | 1 | 1 |
| Mahmudiono et al. [141] | 2017 | 8 | High quality | 1 | 1 | 1 | 1 | 1 | 1 | 1 | 1 |
| Kasaye et al. [144] | 2019 | 6 | Medium quality | 1 | 1 | 1 | 1 | 0 | 9 | 1 | 1 |
| Paul et al. [145] | 2022 | 8 | High quality | 1 | 1 | 1 | 1 | 1 | 1 | 1 | 1 |
| Naz et al. [146] | 2020 | 6 | Medium quality | 1 | 1 | 1 | 1 | 0 | 9 | 1 | 1 |
| Ndagijimana et al. [147] | 2022 | 6 | Medium quality | 1 | 1 | 1 | 1 | 0 | 9 | 1 | 1 |
| Mengesha et al. [148] | 2021 | 6 | Medium quality | 1 | 1 | 1 | 1 | 0 | 9 | 1 | 1 |
| Mann et al. [149] | 2021 | 6 | Medium quality | 1 | 1 | 1 | 1 | 0 | 9 | 1 | 1 |
| Asgary et al. [150] | 2015 | 8 | High quality | 1 | 1 | 1 | 1 | 1 | 1 | 1 | 1 |
| Rahman et al. [151] | 2021 | 6 | Medium quality | 1 | 1 | 1 | 1 | 0 | 9 | 1 | 1 |
| McKenna et al. [152] | 2019 | 8 | High quality | 1 | 1 | 1 | 1 | 1 | 1 | 1 | 1 |
| Joe et al. [153] | 2019 | 6 | Medium quality | 1 | 1 | 1 | 1 | 0 | 9 | 1 | 1 |
| Hossain et al. [154] | 2020 | 6 | Medium quality | 1 | 1 | 1 | 1 | 0 | 9 | 1 | 1 |
| Udoh et al. [155] | 2016 | 6 | Medium quality | 1 | 1 | 1 | 1 | 0 | 9 | 1 | 1 |
| Mugode et al. [156] | 2018 | 8 | High quality | 1 | 1 | 1 | 1 | 1 | 1 | 1 | 1 |
| Bedatu et al. [157] | 2020 | 6 | Medium quality | 1 | 1 | 1 | 1 | 0 | 9 | 1 | 1 |
| Sartika et al. [158] | 2021 | 8 | High quality | 1 | 1 | 1 | 1 | 1 | 1 | 1 | 1 |
| Rahman et al. [159] | 2021 | 8 | High quality | 1 | 1 | 1 | 1 | 1 | 1 | 1 | 1 |
| Boah et al. [161] | 2019 | 8 | High quality | 1 | 1 | 1 | 1 | 1 | 1 | 1 | 1 |
| Campbell et al. [162] | 2017 | 8 | High quality | 1 | 1 | 1 | 1 | 1 | 1 | 1 | 1 |
| Christian et al. [163] | 2020 | 7 | High quality | 1 | 1 | 1 | 1 | 1 | 3 | 1 | 1 |
| Islam et al. [164] | 2019 | 6 | Medium quality | 1 | 1 | 1 | 1 | 0 | 9 | 1 | 1 |
| Gamecha et al. [165] | 2017 | 6 | Medium quality | 1 | 1 | 1 | 1 | 0 | 9 | 1 | 1 |
| Yeshaneh et al. [166] | 2022 | 6 | Medium quality | 1 | 1 | 1 | 1 | 0 | 9 | 1 | 1 |
| Salman et al. [167] | 2020 | 7 | High quality | 1 | 1 | 1 | 1 | 1 | 3 | 1 | 1 |
| Obeyelu et al. [168] | 2021 | 6 | Medium quality | 1 | 1 | 1 | 1 | 0 | 9 | 1 | 1 |
| Sethy et al. [170] | 2014 | 7 | High quality | 1 | 1 | 1 | 1 | 1 | 3 | 1 | 1 |
| Kiarie et al. [171] | 2016 | 6 | Medium quality | 1 | 1 | 1 | 1 | 0 | 9 | 1 | 1 |
| Rajpal et al. [172] | 2020 | 6 | Medium quality | 1 | 1 | 1 | 1 | 0 | 9 | 1 | 1 |
| Humbwavali et al. [173] | 2014 | 6 | Medium quality | 1 | 1 | 1 | 1 | 0 | 9 | 1 | 1 |
| Elnadif [174] | 2020 | 4 | Low quality | 3 | 1 | 1 | 1 | 0 | 9 | 1 | 3 |
| Jude et al. [175] | 2019 | 6 | Medium quality | 1 | 1 | 1 | 1 | 0 | 9 | 1 | 1 |
| Manyong et al. [176] | 2021 | 6 | Medium quality | 1 | 1 | 1 | 1 | 0 | 9 | 1 | 1 |
| Haq et al. [177] | 2022 | 7 | High quality | 1 | 1 | 1 | 1 | 1 | 3 | 1 | 1 |
| Yani et al. [178] | 2019 | 7 | High quality | 1 | 1 | 1 | 1 | 1 | 3 | 1 | 1 |
| Masibo et al. [179] | 2022 | 6 | Medium quality | 1 | 1 | 1 | 1 | 0 | 9 | 1 | 1 |
| Baye et al. [180] | 2022 | 6 | Medium quality | 1 | 1 | 1 | 1 | 0 | 9 | 1 | 1 |
| Anato [181] | 2021 | 6 | Medium quality | 1 | 1 | 1 | 1 | 0 | 9 | 1 | 1 |
| Zembe-Mkabile et al. [182] | 2019 | 7 | High quality | 1 | 1 | 1 | 1 | 1 | 3 | 1 | 1 |
| Parra et al. [183] | 2018 | 6 | Medium quality | 1 | 1 | 1 | 1 | 0 | 9 | 1 | 1 |
| Thompson et al. [184] | 2022 | 6 | Medium quality | 1 | 1 | 1 | 1 | 0 | 9 | 1 | 1 |
| Lassi et al. [185] | 2020 | 6 | Medium quality | 1 | 1 | 1 | 1 | 0 | 9 | 1 | 1 |
| Miller et al. [187] | 2022 | 6 | Medium quality | 1 | 1 | 1 | 1 | 0 | 9 | 1 | 1 |
| Gordon et al. [188] | 2020 | 6 | Medium quality | 1 | 1 | 1 | 1 | 0 | 9 | 1 | 1 |
| Garemo et al. [189] | 2019 | 6 | Medium quality | 1 | 1 | 1 | 1 | 0 | 9 | 1 | 1 |
| Belizario et al. [190] | 2020 | 6 | Medium quality | 1 | 1 | 1 | 1 | 0 | 9 | 1 | 1 |
| Elduma [191] | 2015 | 6 | Medium quality | 1 | 1 | 1 | 1 | 0 | 9 | 1 | 1 |
| Denney et al. [192] | 2019 | 6 | Medium quality | 1 | 1 | 1 | 1 | 0 | 9 | 1 | 1 |
| Tariku et al. [193] | 2020 | 6 | Medium quality | 1 | 1 | 1 | 1 | 0 | 9 | 1 | 1 |
| Danaei et al. [194] | 2015 | 6 | Medium quality | 1 | 1 | 1 | 1 | 0 | 9 | 1 | 1 |
| Vieira et al. [195] | 2015 | 6 | Medium quality | 1 | 1 | 1 | 1 | 0 | 9 | 1 | 1 |
| Grajeda et al. [196] | 2017 | 7 | High quality | 1 | 1 | 1 | 1 | 1 | 3 | 1 | 1 |
| Tariq et al. [197] | 2022 | 6 | Medium quality | 1 | 1 | 1 | 1 | 0 | 9 | 1 | 1 |
| Otsuka et al. [198] | 2015 | 6 | Medium quality | 1 | 1 | 1 | 1 | 0 | 9 | 1 | 1 |
| Engidaye et al. [200] | 2019 | 6 | Medium quality | 1 | 1 | 1 | 1 | 0 | 9 | 1 | 1 |
| Savanur et al. [201] | 2022 | 7 | High quality | 1 | 1 | 1 | 1 | 1 | 3 | 1 | 1 |
| Wali et al. [202] | 2022 | 8 | High quality | 1 | 1 | 1 | 1 | 1 | 1 | 1 | 1 |
| Ferreira et al. [203] | 2019 | 6 | Medium quality | 1 | 1 | 1 | 1 | 0 | 9 | 1 | 1 |
| Perdono et al. [204] | 2021 | 6 | Medium quality | 1 | 1 | 1 | 1 | 0 | 9 | 1 | 1 |
| Asoba et al. [205] | 2020 | 8 | High quality | 1 | 1 | 1 | 1 | 1 | 1 | 1 | 1 |
| Tafese et al. [206] | 2015 | 6 | Medium quality | 1 | 1 | 1 | 1 | 0 | 9 | 1 | 1 |
| Ekholuenetale et al. [207] | 2022 | 6 | Medium quality | 1 | 1 | 1 | 1 | 0 | 9 | 1 | 1 |
| Demmer et al. [208] | 2019 | 8 | High quality | 1 | 1 | 1 | 1 | 1 | 1 | 1 | 1 |
| Di Cesare et al. [209] | 2016 | 7 | High quality | 1 | 1 | 1 | 1 | 1 | 3 | 1 | 1 |
| Oliphant et al. [211] | 2019 | 7 | High quality | 1 | 1 | 1 | 1 | 1 | 3 | 1 | 1 |
| Choudhury et al. [212] | 2016 | 6 | Medium quality | 1 | 1 | 1 | 1 | 0 | 9 | 1 | 1 |
| yang et al. [213] | 2018 | 8 | High quality | 1 | 1 | 1 | 1 | 1 | 1 | 1 | 1 |
| Ayana et al. [214] | 2020 | 6 | Medium quality | 1 | 1 | 1 | 1 | 0 | 9 | 1 | 1 |
| Yim et al. [215] | 2021 | 6 | Medium quality | 1 | 1 | 1 | 1 | 0 | 9 | 1 | 1 |
| Tonguet-Papucci et al. [216] | 2021 | 8 | High quality | 1 | 1 | 1 | 1 | 1 | 1 | 1 | 1 |
| Tasnim et al. [217] | 2014 | 6 | Medium quality | 1 | 1 | 1 | 1 | 0 | 9 | 1 | 1 |
| Gatica-Dominguez et al. [218] | 2017 | 6 | Medium quality | 1 | 1 | 1 | 1 | 0 | 9 | 1 | 1 |
| Richard et al. [219] | 2019 | 7 | High quality | 1 | 1 | 1 | 1 | 1 | 3 | 1 | 1 |
| Alebel et al. [220] | 2022 | 7 | High quality | 1 | 1 | 1 | 1 | 1 | 3 | 1 | 1 |
| Gebremedhin [221] | 2019 | 6 | Medium quality | 1 | 1 | 1 | 1 | 0 | 9 | 1 | 1 |
| Gausman et al. [222] | 2017 | 6 | Medium quality | 1 | 1 | 1 | 1 | 0 | 9 | 1 | 1 |
| Rivadeneira et al. [223] | 2018 | 6 | Medium quality | 1 | 1 | 1 | 1 | 0 | 9 | 1 | 1 |
| Silveira et al. [224] | 2019 | 6 | Medium quality | 1 | 1 | 1 | 1 | 0 | 9 | 1 | 1 |
| Lagares et al. [225] | 2019 | 7 | High quality | 1 | 1 | 1 | 1 | 1 | 3 | 1 | 1 |
| Wali et al. [202] | 2022 | 8 | High quality | 1 | 1 | 1 | 1 | 1 | 1 | 1 | 1 |
| Fenske et al. [226] | 2019 | 7 | High quality | 1 | 1 | 1 | 1 | 1 | 3 | 1 | 1 |
| Ramos et al. [227] | 2017 | 7 | High quality | 1 | 1 | 1 | 1 | 1 | 3 | 1 | 1 |
| Dorsey et al. [228] | 2018 | 6 | Medium quality | 1 | 1 | 1 | 1 | 0 | 9 | 1 | 1 |
| Hanna [229] | 2020 | 8 | High quality | 1 | 1 | 1 | 1 | 1 | 1 | 1 | 1 |
| Mutisya et al. [230] | 2017 | 6 | Medium quality | 1 | 1 | 1 | 1 | 0 | 9 | 1 | 1 |
| Ginsburg et al. [232] | 2018 | 6 | Medium quality | 1 | 1 | 1 | 1 | 0 | 9 | 1 | 1 |
| Rajoo et al. [233] | 2021 | 6 | Medium quality | 1 | 1 | 1 | 1 | 0 | 9 | 1 | 1 |
| Sanchez et al. [234] | 2017 | 7 | High quality | 1 | 1 | 1 | 1 | 1 | 3 | 1 | 1 |
| Scharf et al. [234] | 2013 | 6 | Medium quality | 1 | 1 | 1 | 1 | 0 | 0 | 1 | 1 |
| Kansu et al. [235] | 2019 | 7 | High quality | 1 | 1 | 1 | 1 | 1 | 3 | 1 | 1 |
| Akseer et al. [236] | 2019 | 7 | High quality | 1 | 1 | 1 | 1 | 1 | 3 | 1 | 1 |
| Prasad et al. [237] | 2020 | 6 | Medium quality | 1 | 1 | 1 | 1 | 0 | 9 | 1 | 1 |
| Saeidlou et al. [239] | 2014 | 4 | Low quality | 3 | 1 | 1 | 1 | 1 | 0 | 3 | 0 |
| Saeidlou et al. [240] | 2014 | 5 | Medium quality | 1 | 1 | 3 | 1 | 0 | 9 | 1 | 1 |
| Vonaesch et al. [241] | 2021 | 8 | High quality | 1 | 1 | 1 | 1 | 1 | 1 | 1 | 1 |
| Dipasquale et al.[242] | 2017 | 8 | High quality | 1 | 1 | 1 | 1 | 1 | 1 | 1 | 1 |
| Madiba et al.[243] | 2021 | 6 | Medium quality | 1 | 1 | 1 | 1 | 0 | 9 | 1 | 1 |
| Hartono et al.[245] | 2020 | 6 | Medium quality | 1 | 1 | 1 | 1 | 0 | 9 | 1 | 1 |

**Q1 -** Were the criteria for inclusion in the sample clearly defined? (1= Yes; 0=No; 3=Unclear; 9=Not applicable)

**Q2 -** Were the study subjects and the setting described in detail? (1= Yes; 0=No; 3=Unclear; 9=Not applicable)

**Q3 -** Was the exposure measured in a valid and reliable way? (1= Yes; 0=No; 3=Unclear; 9=Not applicable)

**Q4 -** Were objective, standard criteria used for measurement of the condition? (1= Yes; 0=No; 3=Unclear; 9=Not applicable)

**Q5 -** Were confounding factors identified? (1= Yes; 0=No; 3=Unclear; 9=Not applicable)

**Q6 -** Were strategies to deal with confounding factors stated? (1= Yes; 0=No; 3=Unclear; 9=Not applicable)

**Q7 -** Were the outcomes measured in a valid and reliable way? (1= Yes; 0=No; 3=Unclear; 9=Not applicable)

**Q8 -** Was appropriate statistical analysis used? (1= Yes; 0=No; 3=Unclear; 9=Not applicable)

**Appendix Figure S1.** Pooled prevalence of stunting in developing countries, 2010-22, sub-grouped by year and continent.

**Appendix Figure S2.** Pooled prevalence of wasting in developing countries, 2010-22, sub-grouped by year and continent.

**Appendix Figure S3.** Pooled prevalence of underweight in developing countries, 2010-22, sub-grouped by year and continent.

**Appendix Figure S4**. Funnel plot for prevalence of childhood stunting in developing countries

**Appendix Figure S5**. Funnel plot for prevalence of childhood wasting in developing countries.

**Appendix Figure S6**. Funnel plot for prevalence of childhood underweight in developing countries.
